# A “Clickable” MTX Reagent as a Practical Tool for Profiling Small-Molecule–Intracellular Target Interactions via MASPIT

**DOI:** 10.1002/cmdc.201200493

**Published:** 2013-01-22

**Authors:** Martijn D P Risseeuw, Dries J H De Clercq, Sam Lievens, Ulrik Hillaert, Davy Sinnaeve, Freya Van den Broeck, José C Martins, Jan Tavernier, Serge Van Calenbergh

**Affiliations:** [a] Laboratory for Medicinal ChemistryFaculty of Pharmaceutical Sciences, Ghent University, Harelbekestraat 72, 9000 Gent (Belgium); [b] Cytokine Receptor Laboratory, Department of Medical Protein Research, VIB, Gent and Department of BiochemistryGhent University, Albert Baertsoenkaai 3, 9000 Gent (Belgium); [c] NMR and Structure Analysis Unit, Department of Organic ChemistryGhent University, Krijgslaan 281 S4, 9000 Gent (Belgium)

**Keywords:** click chemistry, MASPIT, methotrexate conjugates, profiling, target identification, three-hybrid

## Abstract

We present a scalable synthesis of a versatile MTX reagent with an azide ligation handle that allows rapid γ-selective conjugation to yield MTX fusion compounds (MFCs) appropriate for MASPIT, a three-hybrid system that enables the identification of mammalian cytosolic proteins that interact with a small molecule of interest. We selected three structurally diverse pharmacologically active compounds (tamoxifen, reversine, and FK506) as model baits. After acetylene functionalization of these baits, MFCs were synthesized via a CuAAC reaction, demonstrating the general applicability of the MTX reagent. In analytical mode, MASPIT was able to give concentration-dependent reporter signals for the established target proteins. Furthermore, we demonstrate that the sensitivity obtained with the new MTX reagent was significantly stronger than that of a previously used non-regiomeric conjugate mixture. Finally, the FK506 MFC was explored in a cellular array screen for targets of FK506. Out of a pilot collection of nearly 2000 full-length human ORF preys, FKBP12, the established target of FK506, emerged as the prey protein that gave the highest increase in luciferase activity. This indicates that our newly developed synthetic strategy for the straightforward generation of MFCs is a promising asset to uncover new intracellular targets using MASPIT cellular array screening.

## Introduction

As we move toward systems biology and personalized medicine, it will become increasingly important to profile small molecule–target interactions and to map this information with metabolic and signaling pathways. Indeed, many clinically used drugs have been found to be more promiscuous than originally thought. However, modulation of multiple targets can also cause harmful side effects, and another considerable challenge is to uncover the mechanisms of toxicities that are not directly related to the desired pharmacological effects of drugs (“off-target pharmacology”). As classical in vitro target profiling requires time- and budget-consuming expression, purification, and assay setup for each individual target, it usually involves testing of a compound against a limited panel of related targets and is thus not comprehensive.

The number of “tried-and-true” drug targets is quite small.[1,2] The emergence of molecular biology and the completion of the human genome project have hitherto failed to produce the expected flood of compounds aimed at new targets. Unbiased, phenotype-based screens represent a promising approach to uncover drugs with a novel mechanism of action. For small molecules discovered in such screens, identifying the biological targets remains largely an ad hoc affair. Traditional approaches using affinity pull-down reagents[3] have been successful for the identification of new targets and have, for example, been recently employed to uncover targets involved in the teratogenic effects of thalidomide.[4] However, sensitivity can be limited, particularly for compounds that exhibit low binding affinity toward their target or for targets expressed at low levels. In these cases, the target protein is lost during the washing steps, or its binding is obscured by the presence of highly abundant (non-specifically binding) proteins.[5] A systematic, widely applicable, and robust approach is badly needed.

**Figure 1:**
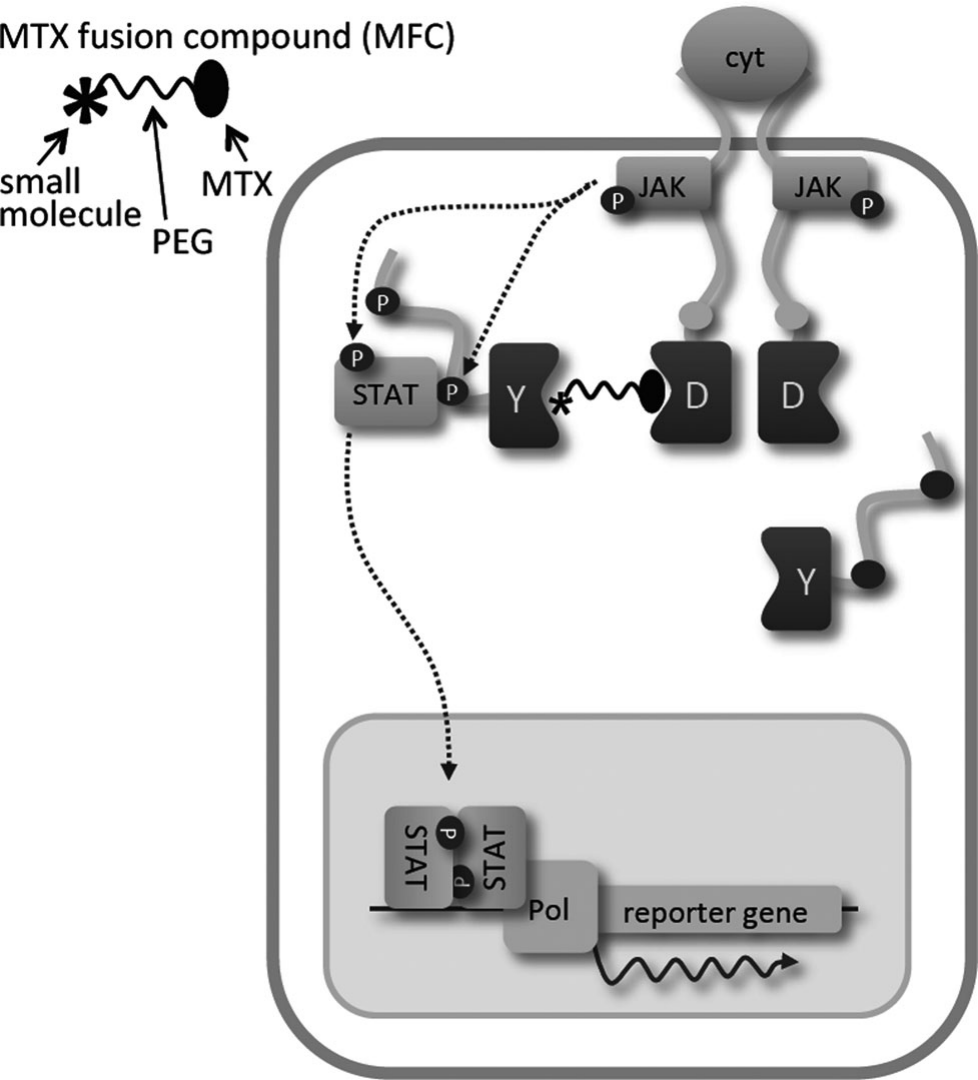
Outline of the MASPIT system. Mammalian cells express a signaling-deficient cytokine receptor containing mutated STAT3 recruitment sites (grey dots), which is fused to DHFR (D). Upon addition of an MFC, which is readily taken up by the cells, the MTX moiety binds to DHFR with high affinity, resulting in the small organic molecule being displayed as bait. A second hybrid polypeptide expressed in the cells consists of a prey protein (Y) coupled to a gp130 cytokine receptor fragment that contains functional STAT3 docking sites (black dots). Physical interaction between the bait small molecule and the prey protein brings the cytokine receptor fragments into close proximity, reconstituting a functional cytokine receptor system. When these cells are stimulated with the appropriate cytokine ligand (cyt), constitutively associated JAK2 kinases are activated, leading to phosphorylation of tyrosine molecules in the gp130 moiety (P) and resulting in the recruitment of STAT3 transcription factors. Subsequently, these STATs are activated through phosphorylation (P) by the activated JAKs. Finally, activated STAT3 complexes migrate to the nucleus as dimers, where they induce expression of a STAT3dependent reporter gene.

MASPIT (mammalian small molecule protein interaction trap) is a three-hybrid trap variant of the original MAPPIT concept[6,7] for the detection of small molecule–protein interactions. MASPIT makes use of a signaling-deficient cytokine receptor lacking STAT3 recruitment sites, which is fused to dihydrofolate reductase (DHFR) (Figure 1). Fusion compounds consisting of an organic molecule of interest tethered to methotrexate (MTX) bind DHFR with very high affinity, allowing presentation of the organic molecule as “bait”. Binding of a chimeric “prey” protein containing functional STAT3 binding sites on the MTX fusion compound (MFC) complements the STAT3 signaling cascade. Hence, ligand binding to the receptor will lead to activation of receptor-associated JAK2 kinases, followed by tyrosine phosphorylation of the STAT3 recruitment motifs of the prey chimeras. Subsequent binding and activation of STAT3 is then easily measured using a STAT3-responsive reporter gene.

MASPIT can be used both analytically, to study designated small molecule–protein interactions, and in searches for interaction partners. Since 2006, our research group has been involved in a large-scale human interactome mapping program.[8,9] As a consequence, a large portion of the human ORFeome is being transferred into MASPIT prey vectors, currently encompassing more than 12 000 ORFs.[10] To optimize screening, a cellular array screening platform was developed.[11] In brief, each prey plasmid from the collection, together with a luciferase reporter construct, was mixed with a transfection reagent to generate prey arrays in 384-well plates. After reverse transfection with a cell pool expressing the receptor–DHFR chimera and addition of the bait MFC, followed by ligand-induced activation of the system, positive interactors were detected simply by measuring the activity of a STAT3-dependent luciferase reporter gene.

In contrast to classical target-based profiling, this mammalian three-hybrid system can provide information regarding unanticipated small molecule–target protein interactions. Another important advantage of MASPIT is the fact that the interactions between small molecules and target proteins occur in living mammalian cells rather than in vitro. Consequently, this might reveal potential effects of post-translational modifications of the target or of the target’s association with additional proteins or other intracellular molecules on small molecule binding.

A necessary component of successful MASPIT applications, however, is the synthesis of appropriate MFCs. Structure–activity relationship (SAR) studies of MTX derivatives have emphasized the importance of selective conjugation to the γ-carboxylic acid of the glutamate moiety to ensure high affinity binding to DHFR through interaction of this enzyme with the free α-carboxylic acid.[12] Hence, an objective of this study was to synthesize a versatile MTX-based building block and explore its use for easy and straightforward ligation to bait small molecules of interest.

## Results and Discussion

To swiftly access a wide variety of MFCs with minimal effort, we envisaged the synthesis of a general MTX conjugate appropriately equipped with a ligation handle. It was estimated that a copper-catalyzed 3+2 azide alkyne cycloaddition (CuAAC)[13] would be a suitable method for the attachment of the small molecule baits to the MTX-linker conjugates, given the high chemoselectivity, mild reaction conditions, and high tolerance for a wide diversity of reaction solvents.

**Scheme 1:**
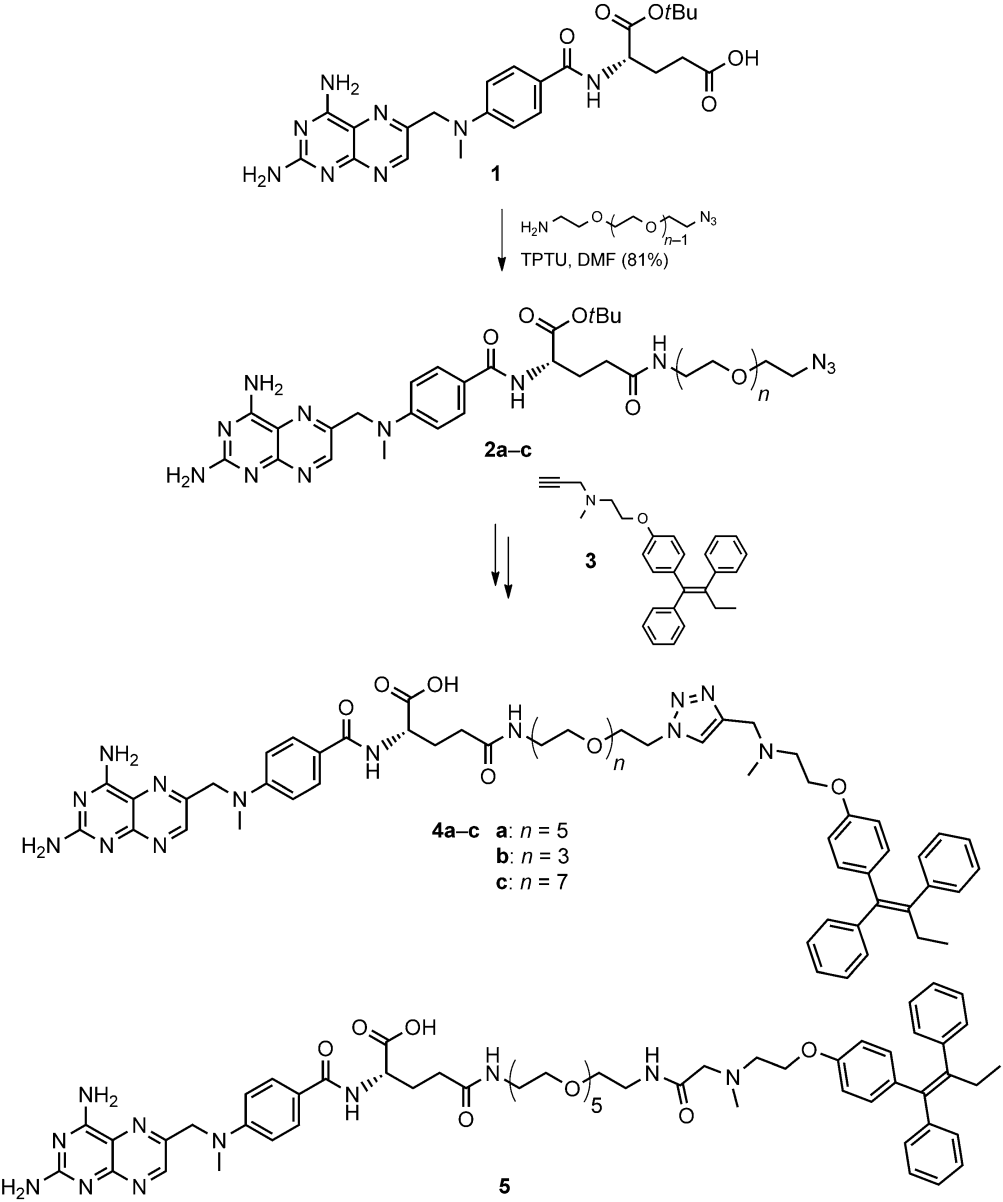
Regioselective synthetic approach to a general MTX conjugate with a terminal azido group as a ligation handle.

A terminal azido group was selected as a ligation handle. To discourage steric hindrance of fusion partners, which could cause the MFC to bind suboptimally to DHFR or allow one to overlook targets that might interact with the unconjugated bait, a PEG linker was introduced between the γ-carboxylic acid and the ligation handle to allow optimal interaction with prey chimeras. To circumvent the formation of a mixture of regioisomers, we condensed α-*tert*-butylmethotrexate **1**[14] with a PEG 4azidoamine using TPTU reagent to obtain satisfactory yields (Scheme 1). Three different MTX-azido reagents (**2 a**–**c**) were synthesized which differed with regard to the number of PEG units.

**Figure 2:**
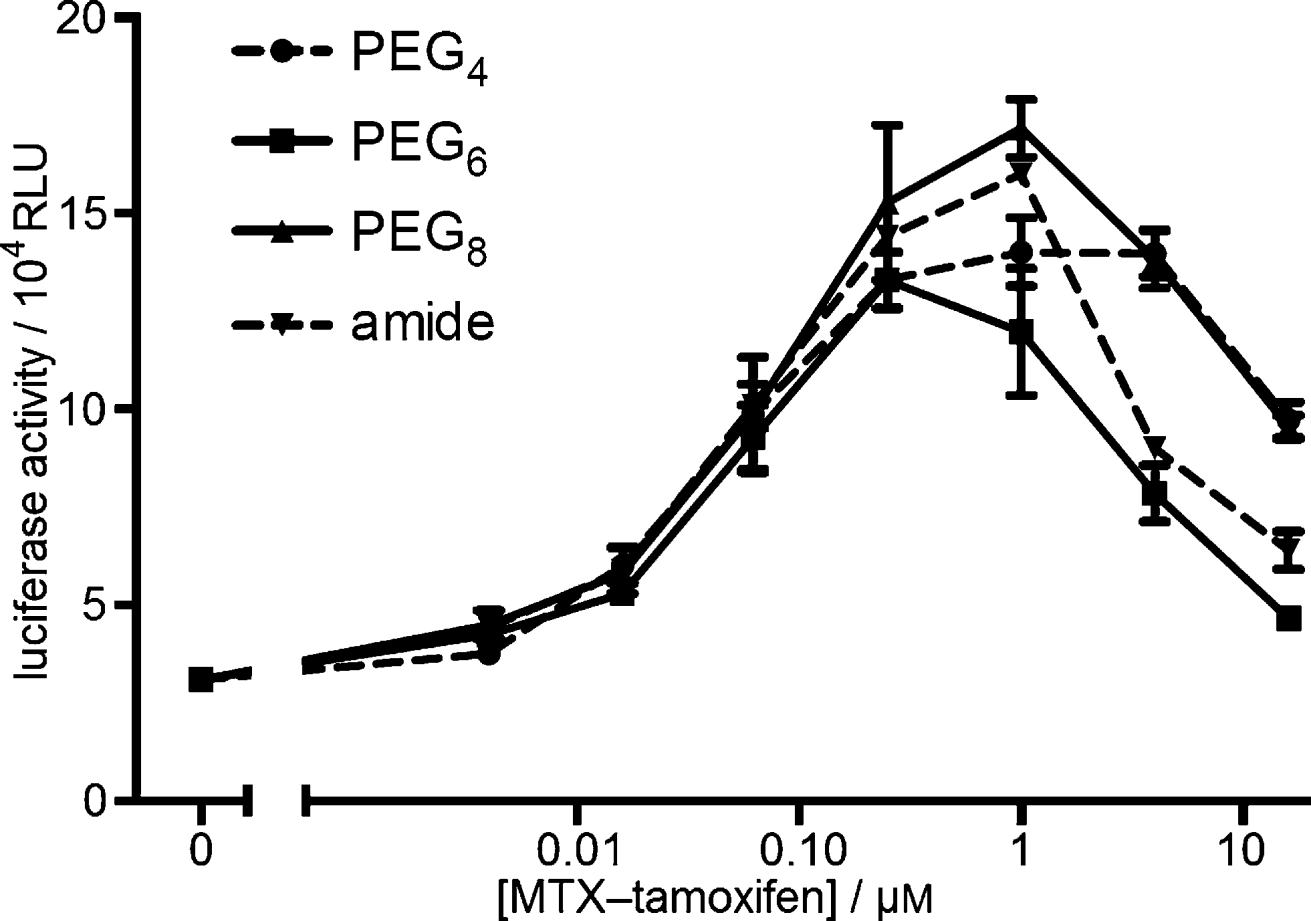
Comparison of different tamoxifen MFCs. Cells were transiently transfected with a pCLG-eDHFR receptor-DHFR plasmid, an ESR1 prey construct, and a luciferase reporter plasmid. They were then treated with combinations of leptin and the indicated concentration of either of the different MTX-tamoxifen fusion compounds: click-coupled through a tetra- (PEG4), hexa- (PEG6), or octaethylene glycol linker (PEG8), or amide-coupled through a hexaethylene glycol linker (amide). The graph shows average luciferase activity of triplicate samples. Error bars represent the standard deviation.

The utility of these MTX-based ligation reagents to form MFCs was demonstrated for three structurally diverse baits of interest, beginning with tamoxifen. The desired tamoxifen–MTX fusion compounds **4 a**–**c** were prepared via click reaction with *N*-desmethyl-*N*-propargyltamoxifen (**3**), which was obtained by propargylation of *N*-desmethyltamoxifen hydrochloride. To investigate the possible influence of the triazole ring on the MASPIT signal, we also prepared an amide-coupled MFC (**5**) from (*N*-desmethyltamoxifen-*N*-yl)acetic acid and an amine MTX ligation reagent obtained from **2 a**. To evaluate tamoxifen–MTX conjugates **4 a**–**c** and **5** in MASPIT, estrogen receptor alpha (ESR1), the established primary target of tamoxifen, was selected as a prey protein. Hence, HEK293T cells were transiently transfected with receptor–DHFR chimera, ESR1 prey constructs, and a STAT3-dependent luciferase reporter gene. Reporter activity was shown to be dependent on MFC concentration, with a similar pattern for all evaluated tamoxifen MFCs and a maximal signal within the 0.1–1 μm range (Figure 2). Although the optimal spacer length may be determined by the nature of the prey, we decided to use a PEG6 linker for the synthesis of two additional MFCs.

**Figure 3:**
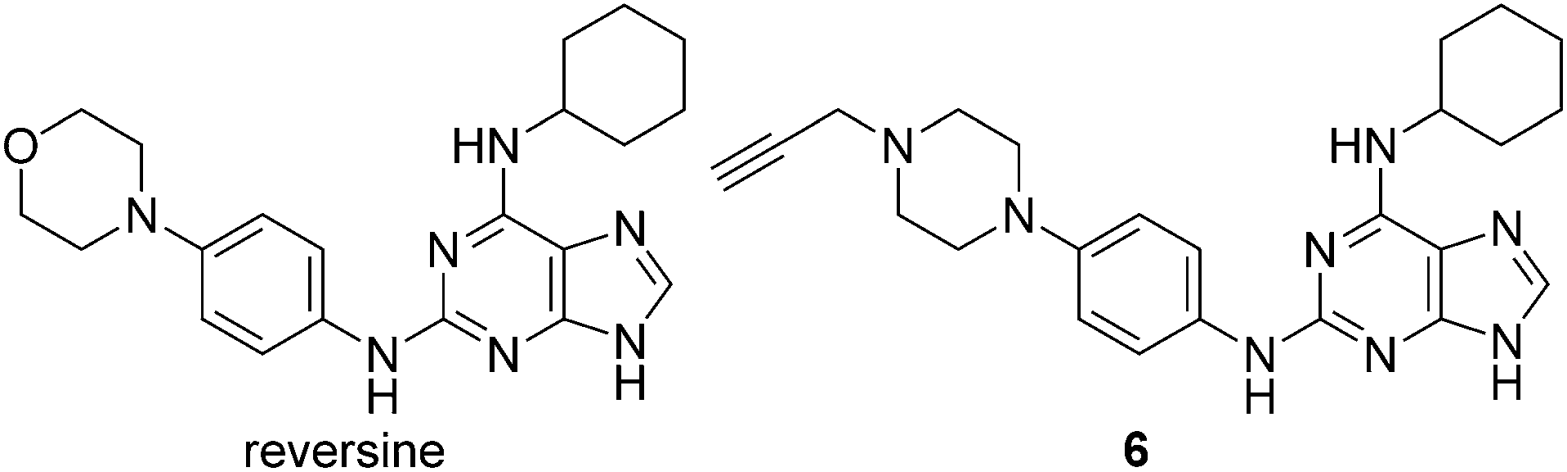
Structure of reversine and an alkynylated analogue used in the synthesis of the MFC.

**Figure 4:**
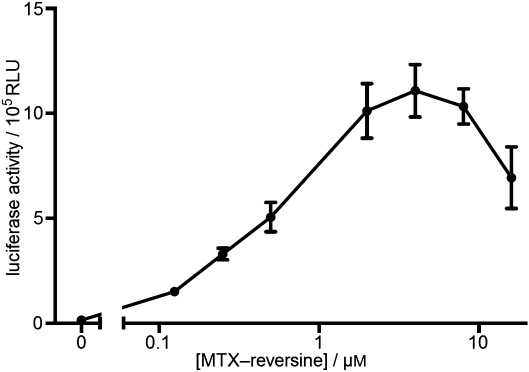
Evaluation of reversine MFC. Cells transfected with a pCLL-eDHFR receptor-DHFR construct, a luciferase reporter plasmid, and a TTK prey plasmid were stimulated with a combination of leptin and the indicated concentration of MFC. The graph shows average luciferase activity of triplicate samples. Error bars represent the standard deviation.

Reversine, a small molecule found to promote dedifferentiation of committed cells into multipotent progenitor-type cells,[15] was selected as a second bait of interest. In vitro inhibition assays on a battery of human mitotic kinases recently indicated that TTK (also known as MPS1) acts as a primary target kinase for reversine (IC_50_=2.8 nm).[16] A click-coupled MFC with reversine was obtained by CuAAC between the alkynylated reversine derivative **6** (Figure 3) and the hydrolyzed **2 a**. Using TTK as a prey plasmid, stimulation with a combination of leptin and MFC gave maximal luciferase activity at a MFC concentration of ∼5 μm (Figure 4).

Tacrolimus (FK506), an immunosuppressant macrolide produced by *Streptomyces tsukubaensis*, was selected as a third model bait. FK506 has found widespread use in organ transplantations as a means to lower the risk of organ rejection. The cellular target of FK506 was identified as peptidyl-prolyl *cis*–*trans* isomerase FKBP12. Binding of FK506 to FKBP12 inhibits calcineurin, a protein phosphatase essential for T cell activation and interleukin expression.[17] FK506 is known to bind to its principal intracellular target, FKBP12, with high affinity,[18] and an extensive amount of structure–activity data is available regarding the binding of FK506 to its receptors, which facilitates selection of the attachment site. FK506 has been modified to create affinity reagents for the isolation and identification of its receptors.[19]

Successful MASPIT profiling of FK506, a highly complex natural product, was pursued to provide clear-cut proof that this system is not confined to evaluation of biologically active compounds that are limited in molecular size and/or complexity. We additionally used this bait to show that selective conjugation of MTX to FK506 via the γ-carboxylic acid offers advantages in readout sensitivity in comparison with the non-regiomeric conjugate mixture previously used. Our final goal was to demonstrate the feasibility of FK506 MFC in identifying protein targets of small molecules using MASPIT in the cellular array assay by screening for proteins that bind to FK506.

**Scheme 2:**
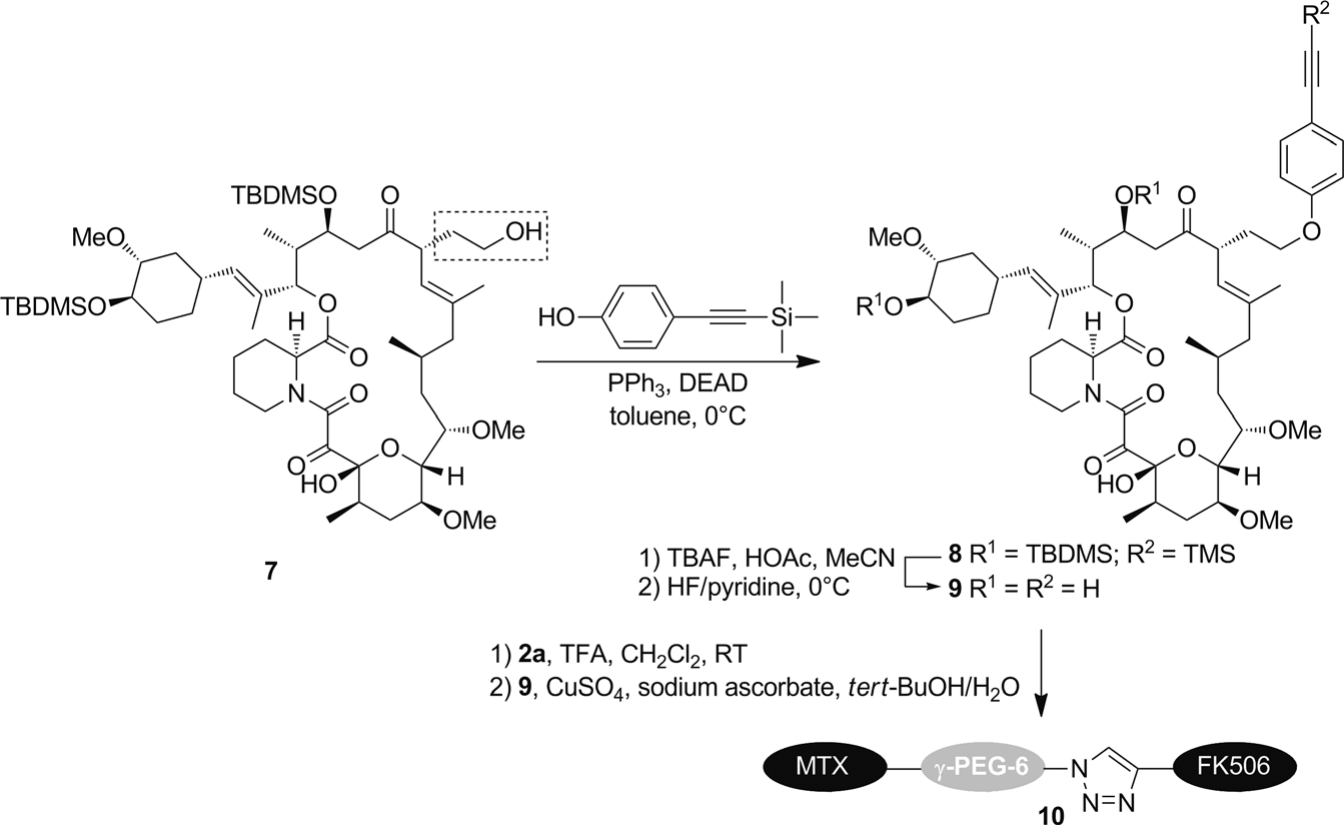
Synthesis of the desired MFC conjugating FK506 to the azido-functionalized MTX using a terminal acetylene graft.

In order to attach FK506 to the azido-functionalized MTX to form the desired MFC, a terminal acetylene had to be grafted onto the macrolide (Scheme 2). The attachment position of this acetylene was meticulously chosen so as to minimize the loss in affinity for FKBP12. Schreiber demonstrated that the allyl moiety of FK506 can be converted into a hydroxyethyl handle (as with compound **7**) without significant loss of activity.[20] Simple alkylation of the primary hydroxy with propargyl bromide under alkaline conditions proceeded with poor regioselectivity, presumably due to the lower p*K*_a_ of the hydroxy group of the ketal functionality. Significantly better regioselectivity was achieved upon reaction of compound **7** with TMS-ethynylphenol[21] under Mitsunobu conditions.[22] Subsequent treatment with TBAF/HOAc and hydrofluoric acid[23] cleanly removed all silyl protecting groups to give acetylene **9**. After removal of the remaining *tert*-butylester of MTX-azide **2 a**, CuAAC with **9** afforded the desired MFC.

**Figure 5:**
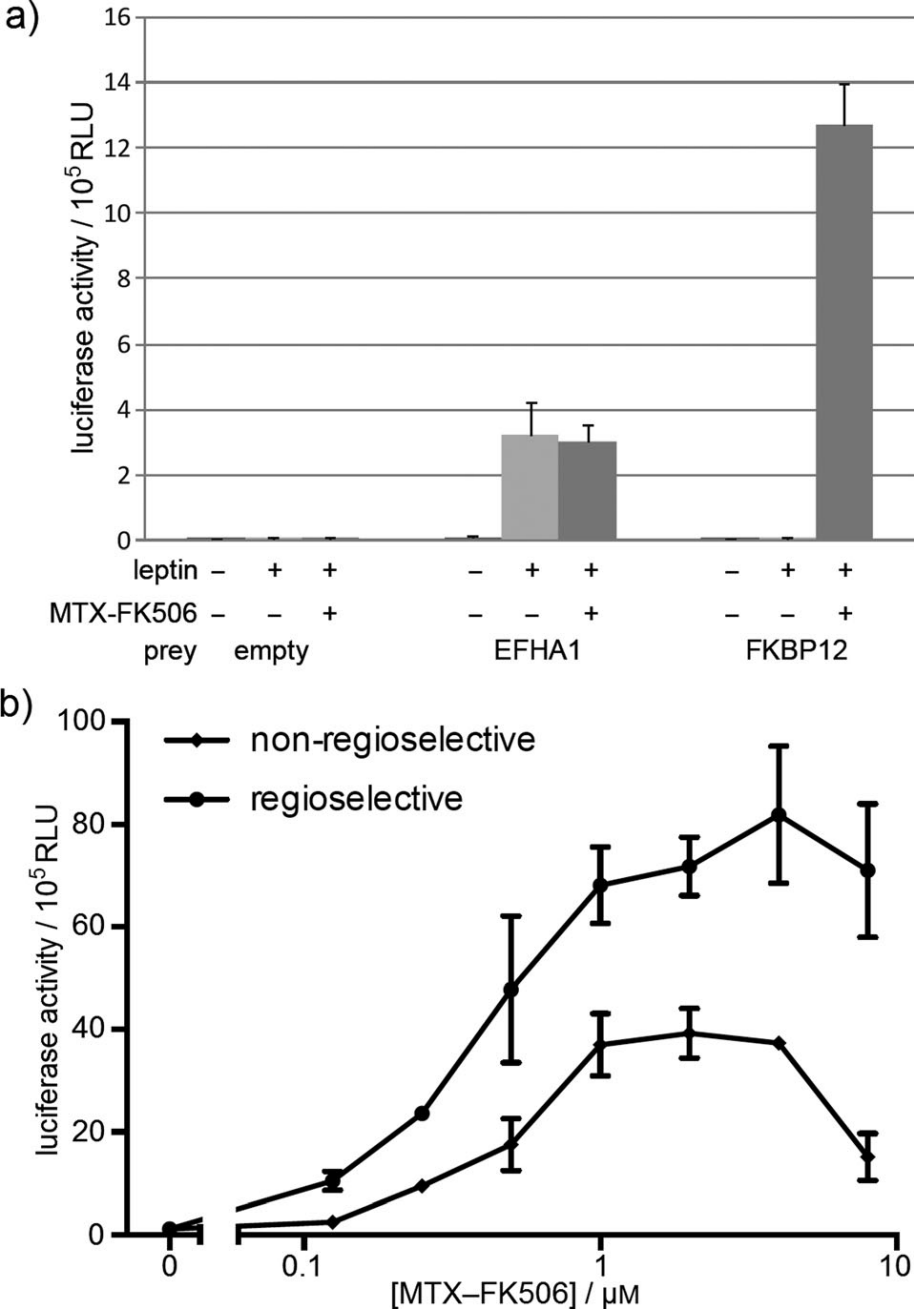
a) Evaluation of the MTX-FK506 conjugate 10 in MASPIT. Cells transfected with a pCLL-eDHFR receptor-DHFR construct, a luciferase reporter plasmid, and either an empty, EFHA1, or FKBP12 prey construct were treated with leptin and/or 1 μm MTX-FK506. Luciferase activity was expressed as relative light units (RLU) and calculated as the average signal of triplicate samples. Error bars represent the standard deviation. b) Comparison of regioselective and non-regiomeric MTX-FK506 conjugate mixtures. Cells transfected with a receptor–DHFR construct (pCLL-eDHFR), FKBP12 prey plasmid, and a luciferase reporter plasmid were treated with leptin and the indicated concentration of either MTX-FK506 conjugate. The graph shows the average luciferase activity of triplicate samples. Error bars represent the standard deviation.

Evaluation of MTX-FK506 conjugate **10** in MASPIT showed that reporter activity was induced only in cells that were treated with both the bait MFC and the cytokine ligand that activates the assay (Figure 5a). No luciferase activity was measured in cells transfected with a combination of the receptor–DHFR chimera with an empty control prey construct. Co-transfection of the receptor–DHFR chimera with a positive control prey that binds to the receptor chimera itself (EFHA1) resulted in bait MFC-independent reporter gene induction.

Next, performance of the γ-substituted MTX-FK506 conjugate was compared with that of a non-regiomeric conjugate mixture (see Supporting Information for details regarding the latter).[24] HEK293T cells expressing receptor–DHFR chimera and FKBP12 prey were treated with the cytokine ligand and a concentration gradient of either of the two bait MFCs (Figure 5b). Clearly, stronger signals were obtained with the regioselective γ-substituted MTX-FK506 fusion compound. This observation was anticipated, as the α-substituted fusion compound, which constituted roughly half of the non-regiomeric conjugate mixture, inhibits formation of the three-hybrid complex necessary for restoration of the functional MASPIT receptor complex, due to its inability to bind to DHFR.

**Figure 6:**
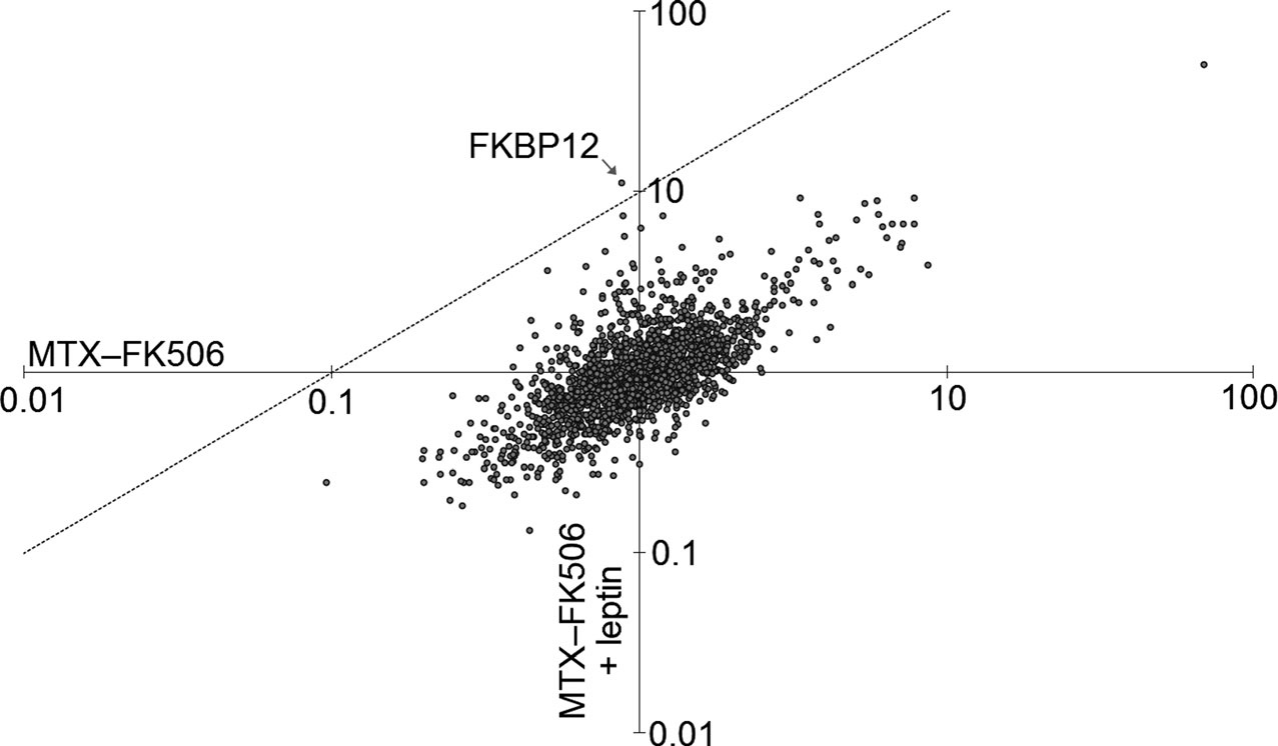
MASPIT cellular array screen with the MTX-FK506 conjugate 10. An array containing 1879 distinct preys was reverse transfected with cells transfected with a receptor-DHFR plasmid (pCLL-eDHFR), and duplicate wells were treated with either 1 μm MTX-FK506 alone or with 1 μm MTX-FK506 combined with leptin. The dot plot shows normalized average luciferase values for each prey. The dashed line indicates the threshold of tenfold induced luciferase activity for MTX-FK506- and leptin-treated over MTX-FK506-treated values. The position of the FKBP12 prey in the plot is indicated.

Having confirmed the functionality of the MTX-FK506 fusion compounds in MASPIT, we next evaluated whether the regioselective MFC **10** could be applied in a cellular array screen for targets of FK506. A pilot collection of nearly 2000 full-length human ORF preys, spotted as transfection mixtures in 384-well microtiter plates,[11] was reverse-transfected with a pool of HEK293T cells transiently transfected with the receptor–DHFR plasmid. Duplicate wells were treated with either MTX-FK506 alone or in combination with the cytokine ligand. The results are shown as a dot plot of normalized luciferase readings for both treatments (Figure 6). Applying a cutoff of tenfold induced luciferase activity for MTX-FK506/ligand-treated over MTX-FK506-treated, the only prey that scored positive corresponds to FKBP12. Importantly, no other previously reported FK506 target proteins were present in the screened collection.

This arrayed screening approach nicely complements the MASPIT cDNA library screening protocol[25]r that has previously been used to search for targets of the kinase inhibitor PD173955. The complementarity of the latter assay lies mainly in the fact that, in contrast to the full-length ORF collection screened by the cellular array assay, a cDNA library also contains partial ORFs encoding protein fragments or domains. Interacting sub-modules in proteins, when isolated from regulatory domains, can allow an interaction to be identified that does not occur in the presence of the regulatory domains. In addition, a cDNA library generally covers a larger portion of the proteome, including multiple protein isoforms for many genes. However, the increased complexity of cDNA libraries, along with the fact that such collections are pooled and not arrayed, makes the screening process much more complicated and time-consuming.

## Conclusions

In conclusion, we have presented a scalable synthesis of a versatile MTX reagent that allows for the rapid synthesis of MFCs compatible with MASPIT from any acetylene-functionalized compound using “click chemistry”. The conjugation methodology, however, is not limited to click chemistry but is also applicable for Staudinger-type ligations or standard peptide coupling conditions. This allows easy and fast access to various MFCs, thereby minimizing the number of chemical manipulations for each construct. The results presented here clearly demonstrate the versatility of the new MTX reagent to generate an MFC of interest for use in MASPIT. Furthermore, we demonstrated the clear benefit of γ-selective functionalization of methotrexate with respect to the signal output. In a cellular array screen, FKBP12 was selectively identified as an interaction partner of FK506, thereby validating the MASPIT system and showing its potential for the identification of the molecular targets responsible for the beneficial or detrimental effects of small molecule drugs.

## Notes

As a service to our authors and readers, this journal provides supporting information supplied by the authors. Such materials are peer reviewed and may be re-organized for online delivery, but are not copy-edited or typeset. Technical support issues arising from supporting information (other than missing files) should be addressed to the authors.
