# Supplementary material for: A “Clickable” MTX Reagent as a Practical Tool for Profiling Small-Molecule–Intracellular Target Interactions via MASPIT
Source: ChemMedChem. 2013 Jan 22;8(3):521–6. doi: 10.1002/cmdc.201200493 (PMC3790973; doi:10.1002/cmdc.201200493)
Supplement: Supplementary file 1 [file cmdc0008-0521-SD1.pdf]

CHEMMEDCHEM
Chemistry & Drug Discovery

Supporting Information

© Copyright Wiley-VCH Verlag GmbH & Co. KGaA, 69451 Weinheim, 2013

A "Clickable" MTX Reagent as a Practical Tool for Profiling Small-Molecule–Intracellular Target Interactions via MASPIT

Martijn D. P. Risseeuw,[a] Dries J. H. De Clercq,[a] Sam Lievens,[b] Ulrik Hillaert,[a] Davy Sinnaeve,[c] Freya Van den Broeck,[c] José C. Martins,[c] Jan Tavernier,*[b] and Serge Van Calenbergh*[a]

cmdc_201200493_sm_miscellaneous_information.pdf

Table of Contents

1. Synthesis S3
   - General S3
   - Synthesis of the amino/azido functionalized polyethyleneglycol spacers S3
   - Studies towards the γ-selective coupling of MTX and compounds **IV** S6
   - Synthesis of tamoxifen-MFCs S12
     - Synthesis of click-coupled tamoxifen MFCs S12
     - Synthesis of amide coupled tamoxifen MFC S16
   - Synthesis of the reversine MFC S21
   - Synthesis of the FK506 MFC S27
2. Molecular biology: MASPIT assay, experimental procedures and results S32
3. Selected NMR spectra S33
   - Compound **2a** S33
   - Compound **4a** S35
   - Compound **5** S39
   - Compound **X** S43
   - Compound **9** S47
   - Compound **10** S48
4. References S49

1 Synthesis

General

All reactions described were performed under an argon atmosphere and at ambient temperature unless stated otherwise. FK506 (Tacrolimus) was purchased from LC laboratories. All other reagents and solvents were purchased from Sigma-Aldrich, Acros Organics or TCI Europe and used as received. Reactions were monitored by TLC analysis using TLC aluminum sheets (Macherey-Nagel, Alugram Sil G/UV_{254}) with detection by spraying with a solution of $(NH_4)_6Mo_7O_{24}$•$4H_2O$ (25 g/L) and $(NH_4)_4Ce(SO_4)_4$•$2H_2O$ (10 g/L) in H_2SO_4 (10%) followed by charring or Ninhydrin (2% in ethanol). Column chromatography was performed on Biosolve 60Å silica gel (32-63 µm). LC-MS analyses were carried out on a Waters Alliance 2695 XE separation Module using a Phenomenex Luna reversed phase C18 column (100 x 2.00 mm, 3 µm) and a water/acetonitrile/formic acid gradient system. High resolution spectra were recorded with a Waters LCT Premier XE Mass spectrometer. ^{1}H- and ^{13}C- NMR spectra were measured on a Varian Mercury-300BB (300/75 MHz), an Avance II Bruker (700/176 MHz) spectrometer equipped with a ^{1}H/^{13}C/^{15}N TXI-Z probe or an Avance III Bruker spectrometer operating at a 1H frequency of 500 MHz, equipped with a BBI probe. Chemical shifts are given in ppm (δ) relative to tetramethylsilane as an internal standard (^{1}H NMR) or $CDCl_3$, CD_3OD or $SO(CD_3)_2$ (^{13}C NMR). Coupling constants are given in Hz. Preparative HPLC purifications were carried out using a Laprep preparative RP-HPLC system equipped with a Xbridge Prep C18 column (19×250 mm, 5 µm) or a Phenomenex Luna C18 column (21.20 x 250 mm, 5 µm) using a water/acetonitrile/formic acid gradient system. Microwave experiments were performed using a Milestone Microsynth under fiberoptic internal temperature control.

Synthesis of the amino/azido functionalized polyethyleneglycol spacers

The synthesis was commenced with the generation of an azide equipped polyethyleneglycol linker. To this end polyethyleneglycols were treated with methanesulfonyl chloride. Treatment of the formed bismesylates **II** with sodium azide yielded diazides **III**. Desymmetrization of these compounds towards the corresponding azidoamines **IV** was carried out in a biphasic system using triphenylphosphine and dilute hydrochloric acid.[i]

Scheme A: Synthesis of the PEG linkers.

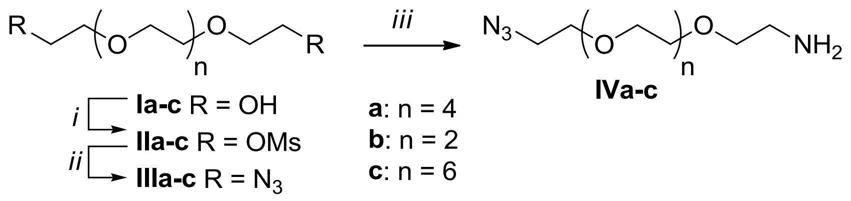

Reagents and conditions: [i] MsCl, triethylamine, CH_2Cl_2, 0°C; [ii] NaN_3, DMF, 60°C, (**a** 91.5%; **b** 97.8%; **c** 92.6% two steps); [iii] first PPh_3, 2.0M HCl, toluene, then NaOH, (**a** 81.0%; **b** 88.2%; **c** 89.1%).

α,ω-diazido,α,ω-dideoxy-hexaethyleneglycol (IIIa)

To an ice cooled solution of hexaethylene glycol (5.0 g, 17.7 mmol) in anhydrous CH_2Cl_2 (30 mL), triethylamine (7.7 mL, 55 mmol) and MsCl (3.1 mL, 40 mmol) were added. The resulting solution was stirred overnight letting the temperature rise to room temperature. The reaction mixture was subsequently poured in 0.5N HCl (100 mL) and after separation of both phases, the aqueous layer was washed with CH_2Cl_2 (3 x 50 mL). The combined organic phases were next washed with sat. $NaHCO_3$ (50 mL) and dried over Na_2SO_4. After filtration and removal of all volatiles under reduced pressure, the crude bismesylate was taken up in DMF (200 mL). Sodium azide (4.0 g, 61.5 mmol) was added and the reaction mixture was stirred overnight at 60°C. The reaction mixture was concentrated *in vacuo.* To the residue were added water (100 mL) and EtOAc (100 ml) and the biphasic mixture was agitated until the residue was completely dissolved and transferred to a separation funnel. The organic layer was separated and the aqueous fraction was washed repeatedly with EtOAc (3 x 100 mL). All EtOAc fractions were pooled, dried on Na_2SO_4 and taken to dryness. The residue was purified by silica gel chromatography (hexane/EtOAc, 1:3 v/v) yielding the product (5.4 g, 16.2 mmol, 91.5%) as a colorless oil. ^{1}H NMR (300 MHz, $CDCl_3$) δ = 3.70-3.65 (m, 20H), 3.38 (t, 4H, *J* = 5.0 Hz); ^{13}C NMR (75 MHz, $CDCl_3$) δ = 70.4-70.3 (wide peak), 69.7, 50.4; HRMS: calcd. for $C_{12}H_{24}N_6O_5Na$ (M+Na): 355.1700, found: 355.1709.

α,ω-diazido,α,ω-dideoxy-tetraethyleneglycol (IIIb)

Tetraethylene glycol (4.9 g, 25.0 mmol) was transformed into the corresponding diazide using the procedure described for compound **IIIa**. Silica gel chromatography (toluene/EtOAc, 1:1 v/v) yielded the title compound (6.0 g, 24.4 mmol, 97.8%) as a slightly yellow liquid. ^{1}H NMR (300 MHz, $CDCl_3$) δ = 3.70-3.67 (m, 12H), 3.39 (t, 4H, *J* = 5.0 Hz); ^{13}C NMR (75 MHz, $CDCl_3$) δ = 70.8 (wide peak), 70.1, 50.7; HRMS: calcd. for $C_8H_{16}N_6O_3Na$ (M+Na): 267.1176, found: 267.1177.

α,ω-diazido,α,ω-dideoxy-octaethyleneglycol (IIIc)

Octaethylene glycol (904 mg, 2.4 mmol) was transformed into the corresponding diazide using the procedure described for compound **IIIa**. Silica gel chromatography (toluene/EtOAc, 15:85 v/v) yielded the title compound (950 mg, 2.3 mmol, 92.6%) as a colorless liquid. ^{1}H NMR (300 MHz, $CDCl_3$) δ = 3.70-3.65 (m, 28H), 3.39 (t, 4H, *J* = 5.0 Hz); ^{13}C NMR (75 MHz, $CDCl_3$) δ = 70.7-70.6 (wide peak), 70.1, 50.7; HRMS: calcd. for $C_{16}H_{32}N_6O_7K$ (M+K): 459.1964, found: 459.1933.

α-Amino,ω-azido,α,ω-dideoxy-hexaethyleneglycol (IVa)

A solution of **IIIa** (5.4 g, 16.2 mmol) in toluene (130 mL) was treated with 2.0M HCl (130 mL) followed by addition of a solution of PPh_3 (4.28 g, 16.3 mmol, 1 eq.) in toluene (15 mL) and the resulting biphasic mixture was vigorously stirred overnight at room temperature. After separation of both phases, the aqueous layer was washed with toluene (3 x 100mL). The aqueous layer was next cooled to 0°C and the pH was adjusted to pH 10 by slow addition of 3N NaOH. The alkaline solution was concentrated *in vacuo* and coevaporated once with toluene. The semisolid residue was extracted with EtOAc (3 x 150 mL). After drying of the combined organic layers over

Na_2SO_4, the residue was purified by column chromatography (CH_2Cl_2/MeOH, 4:1 v/v with 0.25% NH_4OH) affording the title compound (4.2 g, 13.2 mmol, 81%) as a colorless oil.^{1}H NMR (300 MHz, CD_3OD) δ = 3.61-3.68 (m, 18H), 3.53 (t, 2H, *J* = 5.4 Hz), 3.37 (t, 2H, *J* = 5.0 Hz), 2.81 (t, 2H, *J* = 5.4 Hz); ^{13}C NMR (75 MHz, CD_3OD) δ = 72.7, 71.3-71.2 (wide peak), 71.0, 70.8, 51.5, 41.7; HRMS: calcd. for $C_{12}H_{27}N_4O_5$ (M+H): 307.1976, found: 355.1953.

α-Amino,ω-azido,α,ω-dideoxy-tetraethyleneglycol (IVb)

Desymmetrization of diazide **IIIb** (5.9 g, 24.0 mmol) towards the corresponding azidoamine was carried out analogously to the procedure described for compound **IVa**. Silica gel chromatography (CH_2Cl_2/MeOH, 9:1 v/v with 1.0% NH_4OH) yielded the title compound (4.6 g, 21.2 mmol, 88.2%) as a pale yellow oil. ^{1}H NMR (300 MHz, CD_3OD) δ = 4.83-4.80 (m, 2H), 3.68-3.61 (m, 10H), 3.51 (t, 2H, *J* = 5.3 Hz), 3.37 (t, 2H, *J* = 5.0 Hz), 2.78 (t, 2H, *J* = 5.3 Hz); ^{13}C NMR (75 MHz, CD_3OD) δ = 73.4, 71.6-71.1 (wide peak), 51.8, 42.1; HRMS: calcd. for $C_8H_{19}N_4O_3$ (M+H): 219.1452, found: 219.1405.

α-Amino,ω-azido,α,ω-dideoxy-octaethyleneglycol (IVc)

Desymmetrization of diazide **IIIc** (927 mg, 2.2 mmol) towards the corresponding azidoamine was carried out analogously to the procedure described for compound **IVa**, affording the title compound (778 mg, 2.0 mmol, 89.1%) as a pale yellow liquid. ^{1}H NMR (300 MHz, CD_3OD) δ = 4.79-4.77 (m, 2H), 3.69-3.61 (m, 26H), 3.53 (t, 2H, *J* = 5.4 Hz), 3.37 (t, 2H, *J* = 5.0 Hz), 2.80 (t, 2H, *J* = 5.4 Hz); ^{13}C NMR (75 MHz, CD_3OD) δ = 73.1, 71.6-71.1 (wide peak), 51.8, 42.0; HRMS: calcd. for $C_{16}H_{35}N_4O_7$ (M+H): 395.2500, found: 395.2406.

Studies towards the γ-selective coupling of MTX and compounds IV

With the azido-PEG spacers in hand, attention was focused to the γ-selective condensation of MTX to **IV**. Initial condensations using EDC or other standard peptide coupling reagents in DMF consistently provided an intractable mixture of the α- and the γ-amide. Variation of temperature, coupling reagent and solvent had negligible effect on the product distribution. This can be explained by the formation of an acid anhydride in the glutamoyl moiety of MTX which is then opened by the amine of **IV** (Scheme B).

Scheme B: Formation of a regioisomeric mixture of methotrexylamides via an anhydride intermediate.

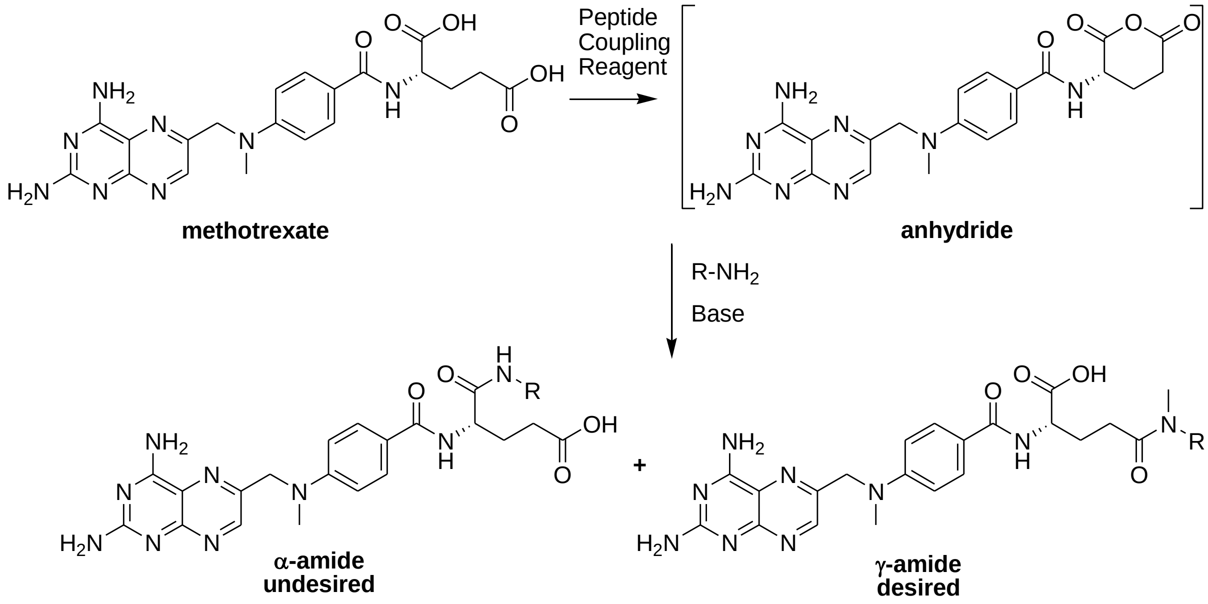

As a result of these difficulties the regioselective synthesis was carried out according to scheme C. Compound **1** was obtained on multigram scale according to a protocol by Francis *et al.*[ii]

Scheme C: γ-Selective synthesis of compounds **2a-c**.

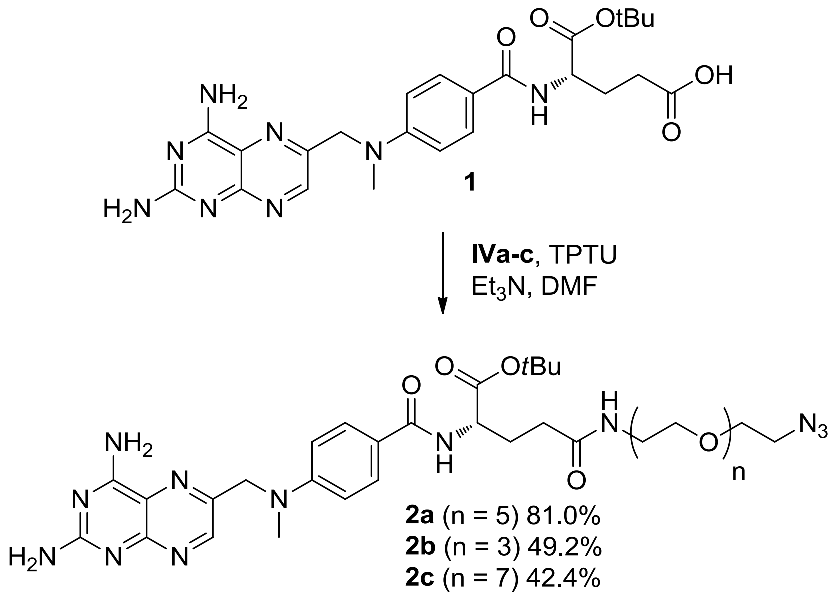

Compound 2a

A solution of methotrexate-α-*tert*-butylester (**1**) (1.02 g, 2 mmol) and aminoazide **IVa** (1.28 g, 4.2 mmol, 2.1 eq.) in DMF was treated with triethylamine (560 µL, 2 mmol, 2 eq.) and TPTU (600 mg, 2 mmol, 1 eq.) and the resulting reaction mixture was stirred for 48 h at room temperature. After removal of the solvent under reduced pressure, the residue was purified by colum chromatography (CH_2Cl_2:MeOH:H_2O, 93:7:0.4). After removal of the solvent under reduced pressure a co-eluting contaminant could be removed by crystallization from MeOH:Et_2O. The title product was an amorphous ochre yellow solid (1.29 g, 81%). HRMS calcd. for $C_{36}H_{55}N_{12}O_9$ (M+H): 799.4210, found: 799.4211.

NMR assignment of MTX-N_3 2a

Nearly complete assignment of the 1H and 13C resonances visible in the spectra could be achieved (see Table 1).

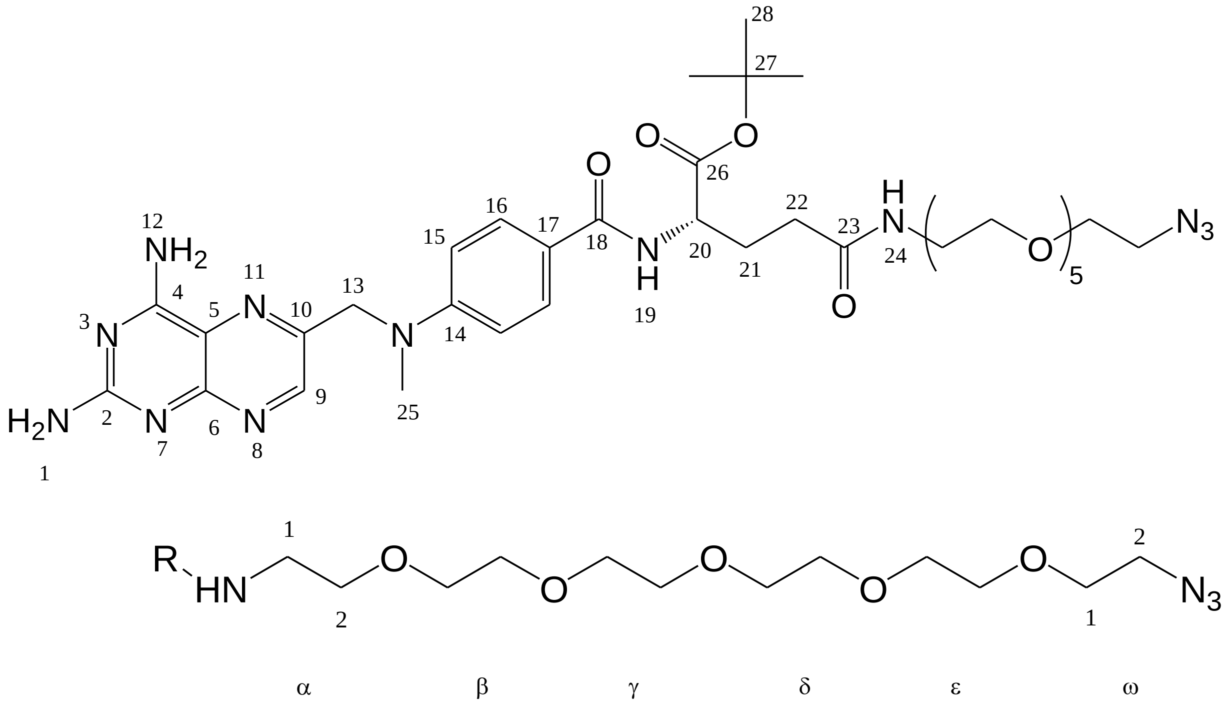

Figure 1: Labelling scheme of MTX-N_3 **2a** and its PEG tail

The methyl resonance of the tBu ester group is easily identified from its integration value; the quaternary carbon is located via HMBC from the tBu protons. This leaves the N-Methyl as the only remaining methyl group to be assigned. The N-Me protons correlate to one Cq of the benzene-like ring, one aromatic CH carbon and the CH_2 carbon on the other side of the N-Me group. In the aromatic region three doublets are visible, only two of which correlate mutually in the COSY, identifying these as belonging to the benzene-like aromatic ring. The third doublet has no directly attached carbon and is therefore the amide of the glutamine-like residue (amide-1). The only triplet in the aromatic region belongs to the other amide (amide-2; no correlation in the HSQC, triplet expected from the neighboring CH_2 group). The remaining singlet in the aromatic region is attributed to the only proton directly attached on the aromatic heterocycle.

These assigned resonances gave sufficient starting points for further assignment as follows.

The amide carbons C_{18}=O (amide 1) and C_{23}=O (amide-2) were identified at 166.20 respectively 171.61 ppm from ${}^{n}J_{CH}$ correlations to their respective amide proton. Amide-1 also correlates to another Cq at 171.51 ppm, which itself correlates to the $C_{20}H\alpha$ proton from the glutamine-like moiety in MTX-N_3. This identifies the Cq as the ester carbonyl carbon C_{26}. The aromatic CH units were discriminated using a set of ${}^{n}J_{CH}$ correlations from which both benzene Cq's could be identified, and later assigned via connections to the N-Me and N-CH_2 protons and the C_{18}=O carbon. The assignment of the aromatic protons was independently confirmed from the relative intensity

and pattern of NOE cross-peaks in the 600 ms NOESY spectrum. Assignment of the heterocycle was less straightforward and remains incomplete. This is due in part to the appearance of the NH_2 protons, which are considerably broadened due to intramolecular exchange phenomena (rotation along the C–NH_2 bond), as well as intermolecular exchange with water (both as seen from the NOESY spectra). As a result, no correlations can be developed to the directly attached (HSQC) or remote (HMBC) carbons from the NH_2 groups. A broadened Cq carbon at 148.71 ppm was tentatively assigned (see table 1).

In all, the above assignments allow to identify all ^{13}C resonances observed above 100 ppm in the ^{13}C APT spectrum. With the exception of the N-Me resonance, all proton resonances between 3 and 4 ppm are contributed by CH_2 type groups, as evidenced by the multiplicity editing in the ^{1}H-^{13}C HSQC spectrum. Only four CH_2 units, labelled a, b, c and d (from left to right) can be separated in this area. Two of these (b and c) are partially overlapping in the ^{1}H spectrum but can clearly be discriminated in the HSQC. Based on the ^{13}C chemicals shift of these 4 CH_2 moieties, two can be attributed to CH_2–O type units (a, c), such as in the PEG chain, and two to CH_2-X type units (b, d) , where X is either the azide or the amide function terminating the PEG chain. From the COSY a is seen to couple with b, while c couples with d, indicating that (a,b) respectively (c,d) are part of the same ethylene unit. Since the amide-2 NH correlates with the carbon directly bound with d, the (c,d) ethylene unit is on the amide side of the PEG-chain, while the (a,b) unit is on the azide side. Thus, the (a,b) unit is identical to the α-ethylene fragment, while the (c,d) unit is identical to the ω-ethylene segment (see labelling scheme). Both CH_2 groups within each of these ethylene moieties were assigned based on chemical shift arguments (CH_2–O) to the highest ppm value.

The remaining proton and carbon resonances are contributed by the PEG chain extending in between the α– and ω–ethylene segments. They extend from 3.54 to 3.44 pm in the ^{1}H spectrum and 69.80 to 69.07 in the ^{13}C spectrum. While some resolution is offered along the ^{13}C dimension, it is insufficient to attempt an assignment of the 4 remaining for PEG-repeat units. The ratio of the total signal intensity of the unresolved ^{1}H area totals 7.93 to 1, which agrees with a total of 4 ethylene fragments, as proposed from the structure.

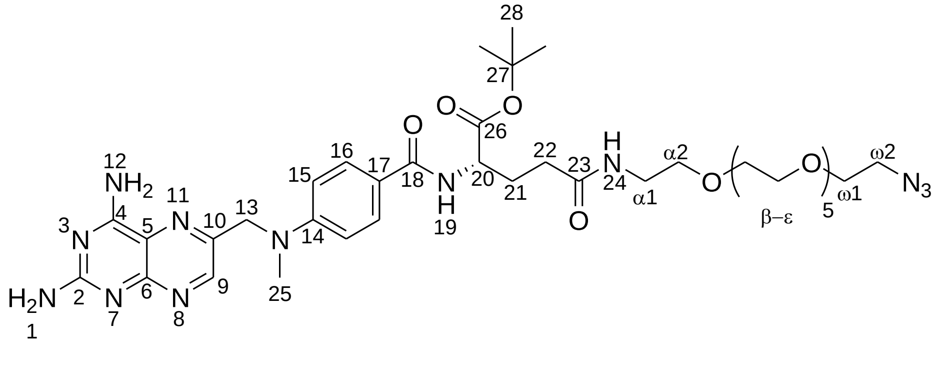

Table 1: *^{1}H and ^{13}C NMR assignment of MTX-N_3 **2a** in DMSO-d_6 at 298K (700/176 MHz).*

Label	^{1}H (ppm)	^{13}C (ppm)	Multiplicity
1 + 12	(8.49/8.27); (7.55/7.09)	n.a.	br s
2	-	n.a.	
4	-	n.a.	
5	-	n.a.	
6	-	148.71	
9	8.63	148.92	S
10	-	n.a.	
13	4.82	54.48	S
14	-	150.77	
15	6.81	111.06	d (8.9 Hz)
16	7.72	128.9	d (8.8 Hz)
17	-	121.27	
18	-	166.2	
19	8.26	-	d (7.25 Hz)
20	4.21	52.99	m
21	a 1.99 / b 1.89	31.77	m
22	2.19	26.46	m
23	-	171.61	
24	7.89	-	t (5.5 Hz)
25	3.22	52.99	s
26	-	171.51	
27	-	80.27	
28	1.38	27.67	s
α1	3.58 (a)	69.22	dd (both ~4.6 Hz)
α2	3.37 (b)	49.97	d (5.15 Hz)
β–δ	3.54 to 3.44	69.80 to 69.07	
ω1	3.36 (c)	69.09	t (5.7 Hz)
ω2	3.16 (d)	38.55	m

Compound 2b

A solution of methotrexate-α-*tert*-butylester (300 mg, 0.59 mmol) and **IVb** (274 mg, 1.26 mmol, 2.1 eq.) in DMF (10 mL) was treated with triethylamine (1.7 mL, 12.2 mmol, 20.7 eq.) and TPTU (299 mg, 1.01 mmol, 1.7 eq.) and the resulting reaction mixture was stirred for 66 h at room temperature. After removal of the solvent under reduced pressure, the residue was purified by silica gel chromatography (CH_2Cl_2:MeOH:Et_3N, 93:7:0.5). A co-eluting contaminant could be removed by crystallization from MeOH:Et_2O yielding the title compound (209 mg, 0.29 mmol, 49.2%) as an amorphous ochre yellow solid. LC-MS: Rt = 6.34 min (10 → 100% MeCN, 15 min run); HRMS: calcd. for $C_{32}H_{47}N_{12}O_7$ (M+H): 711.3685, found: 711.3740.

Compound 2c

A solution of methotrexate-α-*tert*-butylester (300 mg, 0.59 mmol) and **IVc** (548 mg, 1.39 mmol, 2.4 eq.) in DMF (9 mL) was treated with triethylamine (1.7 mL, 12.2 mmol, 20.7 eq.) and TPTU (354 mg, 1.19 mmol, 2.0 eq.) and the resulting reaction mixture was stirred for 71 h at room temperature under nitrogen atmosphere. After removal of the solvent under reduced pressure, the residue was purified by silica gel chromatography (CH_2Cl_2:MeOH:Et_3N, 93:7:0.5). A co-eluting contaminant could be removed by crystallization from MeOH:Et_2O yielding the title compound (222 mg, 0.25 mmol, 42.4%) as an amorphous ochre solid. LC-MS: Rt = 6.52 min (10 → 100% MeCN, 15 min run); HRMS: calcd. for $C_{40}H_{63}N_{12}O_{11}$ (M+H): 887.4734, found: 887.4756.

Synthesis of tamoxifen MFCs

Synthesis of click-coupled tamoxifen MFCs

To acquire an alkyne functionalized analog of tamoxifen, *N*-desmethyltamoxifen hydrochloride[iii] was alkylated with propargyl bromide under alkaline conditions.

Scheme D: Synthesis of alkyne functionalized tamoxifen.

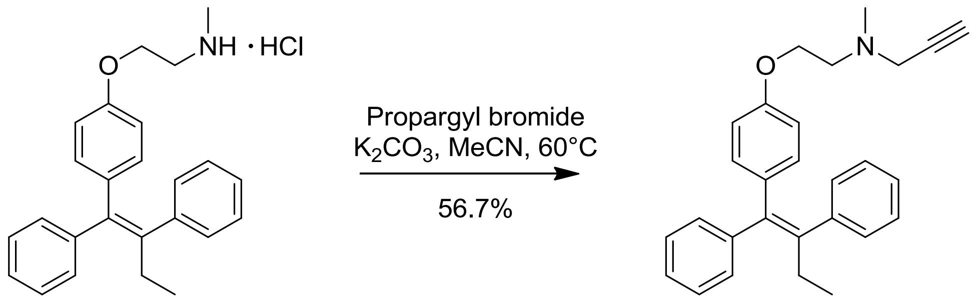

***N*-Desmethyltamoxifen HCl** **3**

***N*-Desmethyl-*N*-propargyltamoxifen (3)**

To a suspension of *N*-desmethyltamoxifen hydrochloride (236 mg, 0.6 mmol) and K_2CO_3 (191 mg, 1.38 mmol) in MeCN (6 mL) was added propargyl bromide (81 µL, 80% in toluene, 0.75 mmol). The reaction mixture was heated to 60°C with vigorous stirring for 18 hours. After cooling to RT, the mixture was concentrated *in vacuo*. The residue was purified by silica gel chromatography (MeOH/CH_2Cl_2, 1:99 v/v) to yield the title compound (136 mg, 0.34 mmol, 56.7%) as a yellow oil. ^{1}H NMR (300 MHz, $CDCl_3$) δ = 7.37-7.07 (m, 10H), 6.76 (d, 2H, *J* = 8.8 Hz), 6.56 (d, 2H, *J* = 8.8 Hz), 3.93 (t, 2H, *J* = 5.8 Hz), 3.40 (d, 2H, *J* = 2.3 Hz), 2.79 (t, 2H, *J* = 5.8 Hz), 2.45 (q, 2H, *J* = 7.4 Hz), 2.36 (s, 3H), 2.20 (t, 1H, *J* = 2.3 Hz), 0.92 (t, 3H, *J* = 7.4 Hz); ^{13}C NMR (75 MHz, $CDCl_3$) δ = 156.8, 143.9, 142.5, 141.5, 138.4, 135.8, 132.0, 129.8, 129.6, 128.2, 128.0, 126.7, 126.1, 113.5, 78.5, 73.5, 65.8, 54.5, 46.1, 42.3, 29.1, 13.7; HRMS: calcd. for $C_{28}H_{30}NO$ (M+H): 396.2322, found: 396.2319.

Conjugate 4a

Azide **2a** (180 mg, 0.23 mmol) was taken up in a mixture of TFA and CH_2Cl_2 (6 mL, 1:1, v/v) and stirred for 40 minutes at RT. The reaction mixture was then taken to dryness, coevaporated twice with toluene and concentrated under high vacuum for 1 hour. The residue was taken up in a mixture of water and *tert*-butanol (2 mL, 1:1, v/v) and alkyne **3** (40 mg, 0.1 mmol), $CuSO_4$ (40 µL, 0.5M, 0.2 eq.) and Na-ascorbate (200 µL, 0.5M, 1.0 eq.) were added. Finally, the resulting reaction mixture was charged with a catalytic amount of TBTA[iv] and heated to 80°C under vigorous stirring. Upon completion of the reaction (96 hours), the solution was cooled to RT and concentrated *in vacuo*. The residue was purified by preparative RP-HPLC (MeCN/water/formic acid 10 → 100%) yielding the title compound (57 mg, 50 µmol, 50.0%) as a pale yellow amorphous solid. LC-MS: Rt = 6.98 min (10 → 100% MeCN, 15 min run); HRMS: calcd. for $C_{60}H_{77}N_{13}O_{10}$ (M+2H): 569.7953, found: 569.7931.

NMR assignment of the tamoxifen part of 4a

For the NMR assignment of the MTX part of **4a**, we refer to the assignment of reversine conjugate **X** (see further). The labelling scheme of the distinct part of **4a** is depicted below:

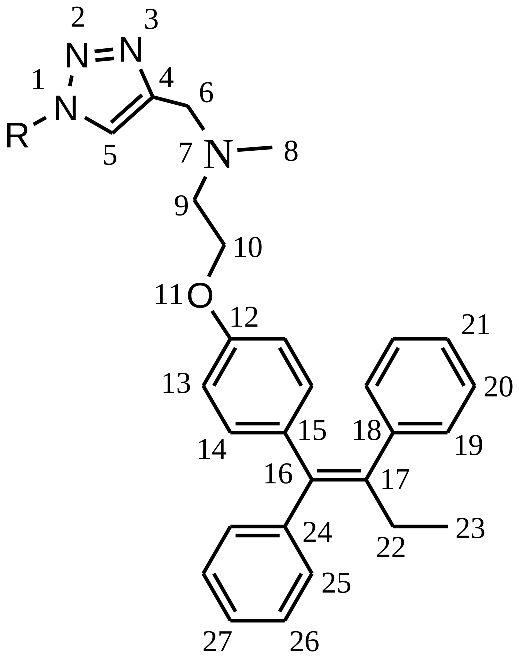

Figure 2: Labeling scheme of the tamoxifen part of **4a**

From the ω2 CH_2 protons in the PEG chain, there is an HMBC correlation to a CH at 124.0 ppm. With HSQC this is seen to correlate to a ^{1}H singlet at 7.92 ppm, this is H-r5. Another HMBC correlation to C-r5 comes from a CH_2 singlet at 3.60 ppm, this is H-r6. Starting from H-r6, two more HMBC correlations are seen, one to a C_q at 143.2 ppm which is assigned to be C-r4 and one to a CH_3 at 42.0 ppm that has an HSQC correlation to a ^{1}H at 2.17 ppm and is assigned to Me-r8. From Me-r8, there are NOESY correlations visible to H-r6, a CH_2 triplet at 2.63 ppm and a CH_2 triplet at 3.91 ppm; these can be assigned to respectively H-r9 and H-r10. H-r10 has an HMBC correlation to a C_q at 156.4 ppm, which is assigned to C-r12. Another HMBC correlation to C-r12 is visible from a CH that integrates for 2 at 6.72 ppm; this is assigned to H-r14. From this signal, there is a COSY correlation to a CH that integrates for 2 at 6.59 ppm, this is H-r13. From H-r13 an HMBC to 156.3 ppm is visible, this is C-r15. Thus a consistent picture emerges for the first aromatic moiety.

In the HSQC there is one more CH_3 signal visible, this is a triplet at 0.84 ppm and can be assigned to the Me-r23. It shows also a COSY correlation to a CH_2 quadruplet at 2.36 ppm, which is H-r22. An HMBC correlation is visible from H-r23 and H-r22 to a C_q at 143.2 ppm, this is C-r17. Another HMBC correlation to C-r17 comes from the left part of a CH type resonance at 7.12 ppm that integrates for 3 ^{1}H's and comes from, according to the HSQC spectrum, two carbons. The left part can be assigned to H-r19. The right part shows an HMBC correlation to the left part and vice versa, we can thus assign the right part to be H-r21. This also agrees with the sum of 3 for this resonance. From these two resonances there is a COSY correlation to the right part of a CH signal at 7.18-7.20 ppm that in total integrates for 4 ^{1}H's; this can be assigned to H-r20. This signal also shows an HMBC correlation to a C_q at 141.7 ppm, C-r18. Thus a consistent picture emerges for the second aromatic moiety. Also an HMBC to

C-r18 is visible from H-r22. Another HMBC correlation from H-r22 goes to a C_q at 137.8 ppm, this is C-r16, also the left part of the signal at 7.18-7.20 ppm shows an HMBC correlation to C-r16 and can thus be assigned to H-r25. H-r25 has a COSY correlation to a CH at 7.37 ppm that integrates for 2 protons and is assigned to H-r26. From this signal, an HMBC correlation is visible to a C_q at 143.2 ppm, this is C-r24. Also from H-r26 a COSY correlation is visible to a CH signal at 7.28 ppm that integrates for 1 proton and is assigned to H-r27. Thus a consistent picture emerges for the third aromatic moiety.

Table 2: *Assignment of **4a** in dmso-d6 at 298K*

Label	^{1}H (ppm)	^{13}C (ppm)	Multiplicity
4	-	143.2	
5	7.92	124.0	s
6	3.62	52.0	s
8	2.17	42.0	s
9	2.63	54.7	t
10	3.91	65.6	t
12	-	156.4	
13	6.59	113.4	d
14	6.72	131.4	d
15	-	156.3	
16	-	137.8	
17	-	140.6	
18	-	141.7	
19	7.12	129.4*	-
20	7.18	128.0*	-
21	7.11	126.3*	-
22	2.36	28.5	q
23	0.84	13.3	t
24	-	143.2	
25	7.20	129.0*	-
26	7.37	128.3	-
27	7.28	126.7	-

* tentative assignment only

Conjugate 4b

Azide **2b** (164 mg, 0.23 mmol) was deprotected and subsequently conjugated to alkyne **3** (40 mg, 0.1 mmol) using the procedure described for conjugate **4a**. Upon completion of the reaction (18 hours) and workup, the material was purified by preparative RP-HPLC (MeCN/water/formic acid 10 → 100%), yielding the title compound (55 mg, 52 µmol, 52.0%) as a pale yellow amorphous solid. LC-MS: Rt = 7.04 min (10 → 100% MeCN, 15 min run); HRMS: calcd. for $C_{56}H_{69}N_{13}O_8$ (M+2H): 525.7691, found: 525.7696.

Conjugate 4c

Azide **2c** (204 mg, 0.23 mmol) was deprotected and subsequently conjugated to alkyne **3** (40 mg, 0.1 mmol) using the procedure described for conjugate **4a**. Upon completion of the reaction (18 hours) and workup, the material was purified by preparative RP-HPLC (MeCN/water/formic acid 10 → 100%), yielding the title compound (78 mg, 63 µmol, 63.4%) as a pale yellow amorphous solid. LC-MS: Rt = 7.11 min (10 → 100% MeCN, 15 min run); HRMS: calcd. for $C_{64}H_{85}N_{13}O_{12}$ (M+2H): 613.8215, found: 613.8204.

Synthesis of amide coupled tamoxifen MFC

Carboxylate functionalized tamoxifen was generated in two steps from *N*-desmethyltamoxifen hydrochloride. Alkylation with methyl bromoacetate followed by saponification of the ester with concomitant neutralization provided the acid.

Scheme E: Synthesis of carboxylate functionalized tamoxifen.

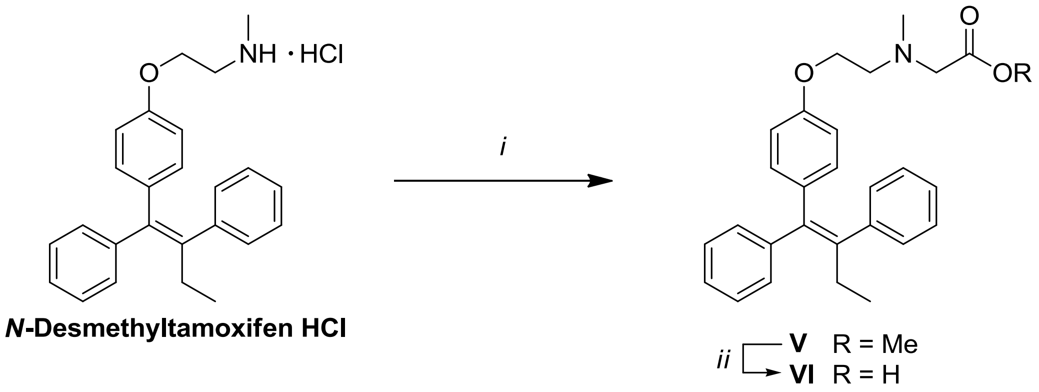

Reagents and conditions: [i] Methyl bromoacetate, DIPEA, CH_2Cl_2, 0°C→RT, 90.5%; [ii] first NaOH, MeOH, 50°C, then HCl, RT (± quant.).

Methyl (*N*-desmethyltamoxifen-*N*-yl)acetate (V)

To an ice cooled solution of methyl bromoacetate (39 µL, 0.42 mmol) in CH_2Cl_2 (1.3 mL), *N*-desmethyltamoxifen hydrochloride (165 mg, 0.42 mmol) and DIPEA (183 µL, 1.05 mmol) were added. The resulting solution was stirred overnight letting the temperature rise to room temperature. The mixture was diluted with CH_2Cl_2 (6.5 mL), washed with sat. $NaHCO_3$ (2 x 10 mL), dried over Na_2SO_4 and concentrated *in vacuo*. The residue was purified by silica gel chromatography (MeOH/CH_2Cl_2, 1:99 v/v) yielding the title compound (161 mg, 0.38 mmol, 90.5%) as a pale white oil. ^{1}H NMR (300 MHz, $CDCl_3$) δ = 7.37-7.07 (m, 10H), 6.76 (d, 2H, *J* = 8.7 Hz), 6.53 (d, 2H, *J* = 8.7 Hz), 3.95 (t, 2H, *J* = 5.6 Hz), 3.64 (s, 3H), 3.37 (s, 2H), 2.90 (t, 2H, *J* = 5.7 Hz), 2.45 (q, 2H, *J* = 7.5 Hz), 2.45 (s, 3H), 0.92 (t, 3H, *J* = 7.4 Hz); ^{13}C NMR (75 MHz, $CDCl_3$) δ = 171.6, 156.7, 143.9, 142.5, 141.5, 138.4, 135.7, 132.0, 129.8, 129.6, 128.2, 128.0, 126.6, 126.1, 113.5, 66.1, 58.6, 55.7, 51.6, 43.2, 29.1, 13.7; HRMS: calcd. for $C_{28}H_{32}NO_3$ (M+H): 430.2377, found: 430.2372.

(*N*-desmethyltamoxifen-*N*-yl)acetic acid (VI)

A solution of compound **V** (249 mg, 0.58 mmol) in MeOH (13 mL) was treated with NaOH (0.96 mL, 4.0M). The resulting reaction mixture was stirred for 6 hours at 50°C. After cooling to RT, the mixture was neutralized by addition of HCl (1.31 mL, 3.0M), concentrated *in vacuo* and coevaporated twice with toluene. The residue was purified by silica gel chromatography (MeOH/CH_2Cl_2, 12:88 v/v) yielding the title compound (342 mg) as a white amorphous solid. The additional weight could be explained by a significant amount of silica gel present in the material. ^{1}H NMR (300 MHz, $CDCl_3$) δ = 7.31-7.06 (m, 10H), 6.72 (d, 2H, *J* = 7.8 Hz), 6.44 (d, 2H, *J* = 8.1 Hz), 4.07 (m,

2H), 3.86 (br s, 2H), 3.46 (m, 2H), 2.77 (br s, 3H), 2.43 (q, 2H, *J* = 7.2 Hz), 0.92 (t, 3H, *J* = 7.4 Hz); LC-MS: Rt = 8.87 min (10 → 100% MeCN, 15 min run); HRMS: calcd. for $C_{27}H_{30}NO_3$ (M+H): 416.2220, found: 416.2065.

With the acid in hand, the MFC was constructed. Methotrexate derivative **2a** was deprotected with trifluoroacetic acid and subsequently reduced using trimethylphosphine. Purification of the crude amine followed by PyBOP mediated condensation to acid **VI** yielded MFC **5** in 32.5% after HPLC purification.

Scheme F: Synthesis of amide linked tamoxifen MFC.

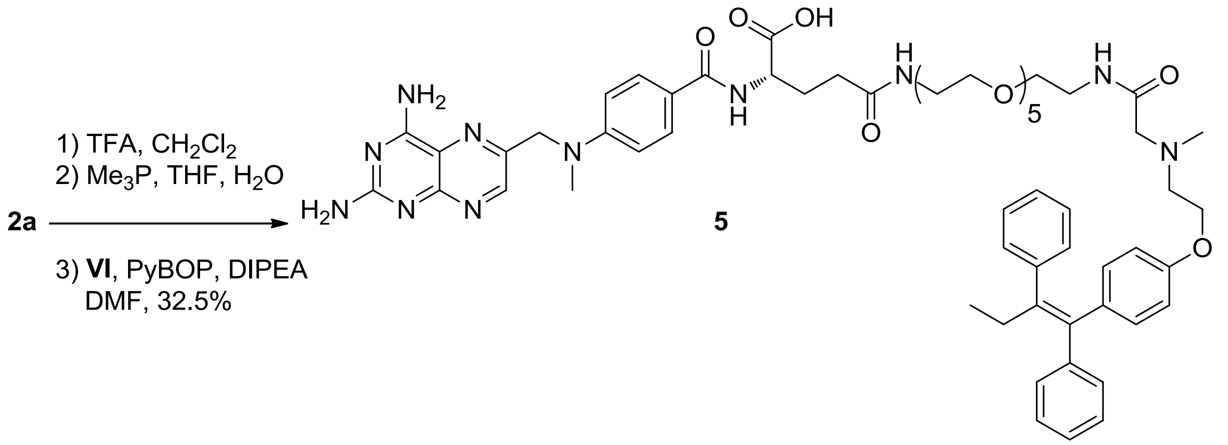

Conjugate 5

Azide **2a** (160 mg, 0.2 mmol) was taken up in a mixture of TFA and CH_2Cl_2 (7 mL, 1:1, v/v) and stirred for 40 minutes at RT. The reaction mixture was then taken to dryness, coevaporated twice with toluene and concentrated under high vacuum for 1 hour. The residue was taken up in THF (4 mL), treated with a solution of Me_3P in THF (2 mL, 1.0M) and stirred overnight at room temperature. Subsequently, H_2O (100 µL) was added and stirring was continued for 1 hour. The solution was concentrated *in vacuo* and the resulting residue was taken up in a mixture of *tert*-butanol and H_2O (4 mL, 1:1, v/v) and lyophilized. The presence of a significant contaminant bearing an additional methyl group, as judged by LC-MS (calcd. for $C_{33}H_{52}N_{10}O_9$ (M+Me+2H): 366.19539, found: 366.1926), necessitated an additional purification. Therefore the crude dry material was purified by preparative RP-HPLC (MeCN/water/formic acid 5 → 100%) yielding the pure amine (41 mg, 57 µmol, 28.5%) as an ochre semi solid. LC-MS: Rt = 11.40 min (5 → 15% MeCN, 18 min run); HRMS: calcd. for $C_{32}H_{50}N_{10}O_9$ (M+2H): 359.18757, found: 359.1852. Acid **VI** (90 mg, ≈ 153 µmol)[v] was taken up in DMF (1.85 mL) and preactivated for 5 minutes using PyBOP (60 mg, 115 µmol) and DIPEA (150 µL, 861 µmol). The MTX-amine (41 mg, 57 µmol) was taken up in DMF (0.5 mL) and combined with the preactivation mixture and stirred for 4 hours. The solution was concentrated *in vacuo* and the residue was purified by preparative RP-HPLC (MeCN/water/formic acid 10 → 100%) yielding the title compound (21 mg, 19 µmol, 32.5%) as a pale yellow amorphous solid. LC-MS: Rt = 7.00 min (10 → 100% MeCN, 15 min run); HRMS: calcd. for $C_{59}H_{77}N_{11}O_{11}$ (M+2H): 557.7897, found: 557.7875.

NMR assignment of the MTX part of 5

The labelling is depicted in the following Figure 4.

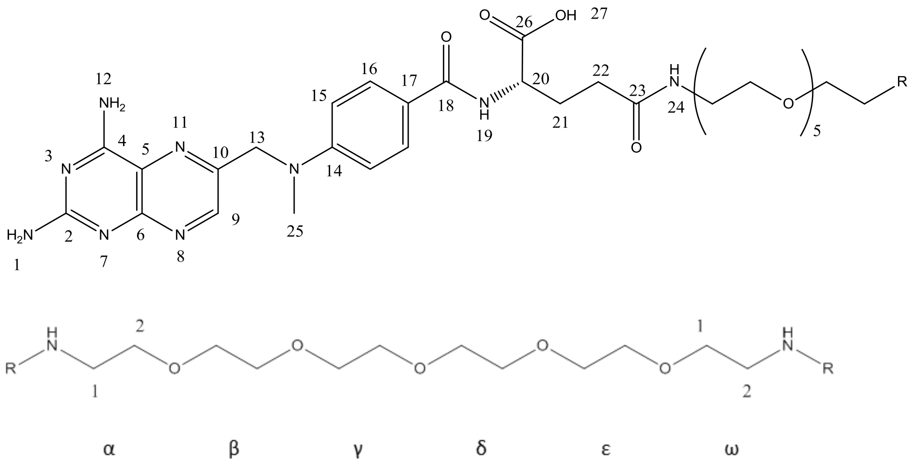

Figure 3: Labelling scheme of the MTX part of MFC **5**

Nearly complete assignment of the ^{1}H and ^{13}C resonances visible in the spectra could be achieved (see Table 3).

Table 3: *Assignment of MTX-R in dmso-d6 at 298K*

Label	^{1}H (ppm)	^{13}C (ppm)	Multiplicity
1 + 12	n.a.	-	
2	-	n.a.	
4	-	n.a.	
5	-	n.a.	
6	-	155.21*	
9	8.55	148.7	s
10	-	145.9	
13	4.76	54.8	s
14	-	150.9	
15	6.81	111.1	d
16	7.69	128.6	d
17	-	121.4*	
18	-	165.8*	
19	8.08	-	s
20	4.18	52.7	q
21	2.00+1.88	27.15	m
22	2.16	32.0	m
23	-	171.9	
24	7.91	-	t
25	3.19	39.05	s
26	-	173.5	
27	n.a.	-	
α1	3.16	38.5	q
α2	3.35	69.0	t
β–ω1	3.4-3.55	69.0-70.4	
ω2	3.18	38.0	

* tentative assignment only

Assignment of the tamoxifen part of the molecule

The labelling scheme of the right part of **5** is depicted below; r is not mentioned in the labelling:

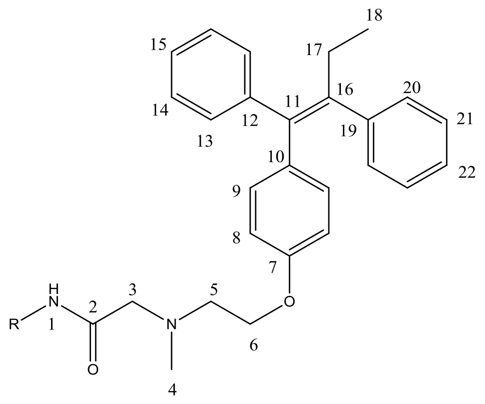

Figure 4: Labelling scheme of the tamoxifen part of **5**

Me-r4 is easily found at 2.26 ppm because it is the only remaining CH_3 group that is a singlet.

Starting from this singlet, there are two HMBC correlations, one to a CH_2 at 55.8 ppm and one to a CH_2 at 61.0 ppm. The CH_2 at 55.8 ppm correlates to a proton resonance at 2.68 ppm that is a triplet and can thus be assigned to H-r5. The other CH_2 has a proton resonance at 2.98 ppm that is a singlet and can be assigned to H-r3. From H-r3 an HMBC correlation is visible to a C_q at 169.7 ppm that is assigned to C-2. From H-r3 and H-r4 a NOESY correlation is visible to a triplet at 7.66 ppm that has no HSQC correlation and can be assigned to H-r1.

From H-r5 there is a COSY correlation visible to a triplet at 3.91 ppm that can be assigned to H-r6. In the HMBC spectrum, a correlation from H-r6 to a C_q at 156.6 ppm is visible, this is assigned to C-7. Another two proton resonances have an HMBC correlation to this last one, an aromatic doublet at 6.6 ppm and one at 6.73 ppm. These also have a COSY correlation to each other and can thus be assigned to H-r9 and H-r8; no distinction can be made between both however. Since we would expect only one to have an HMBC correlation to C-7 and the other one to have an HMBC to C-10, C-10 can tentatively be assigned to be at app. the same resonance as C-7.

Another CH_3 group is present in this part of the molecule, a triplet at 0.82 ppm; this is assigned to H-r18. From this resonance, a COSY correlation is visible to a quadruplet at 2.36 ppm, which is assigned to H-r17. From both H-r18 and H-r17 an HMBC correlation is visible to a C_q at 140.7 ppm, this is C-16.

In the aromatic region there are still 4 signals left that integrate all together for 10 protons. The triplet at 7.37 ppm integrates for 2 protons and is assigned to H-r14. H-r14 has one HMBC correlation to a C_q at 143.2 ppm, which is assigned to C-12, as expected, there are no other HMBC correlations to this quaternary carbon. From H-r14 there are two COSY correlations, one to the triplet at 7.28 ppm, which is assigned to H-r15 and one to the pseudo triplet at 7.19 ppm, which is actually a combination of two doublets and from which the left part can be assigned to H-r13, that has an HMBC correlation to a C_q at 137.9 ppm, C-11. The right part of the pseudo triplet at

7.19 ppm is assigned to H-r21 and has an HMBC correlation to a C_q at 141.8 ppm, C-19. H-r21 also has a COSY correlation to the remaining aromatic resonance at 7.11 ppm that integrates for 3 protons and is assigned to both H-r22 and H-r20, which also shows an HMBC correlation to C-16.

Table 4: *Assignment of the tamoxifen part of **5** in dmso-d6 at 298K*

Label	^{1}H (ppm)	^{13}C (ppm)	Multiplicity
1	7.66	-	t
2	-	169.7	
3	2.98	61.0	s
4	2.26	43.0	s
5	2.68	55.9	t
6	3.91	65.4	t
7	-	156.6	
8 or 9	6.73	131.3	d
8 or 9	6.60	113.4	d
10	-	156.6*	
11	-	137.9	
12	-	143.2	
13	7.19	128.9	d (pseudo triplet)
14	7.37	128.3	t
15	7.28	126.6	t
16	-	140.7	
17	2.36	28.5	q
18	0.82	13.3	t
19	-	141.8	
20	7.12	129.4	m
21	7.18	127.9	d (pseudo triplet)
22	7.11	126.2	m

* tentative assignment only

Synthesis of the reversine MFC

To obtain the alkyne functionalized reversine analog **6**, first *N*-(4-nitrophenyl)piperazine was propargylated under alkaline conditions. Tin(II) mediated reduction[vi] of the nitro moiety gave access to aniline **IX** which could be reacted with N^6-cyclohexyl-2-fluoroadenine[vii] at elevated temperature to obtain compound **6** as a solid.

Scheme G: Synthesis of alkyne functionalized reversine.

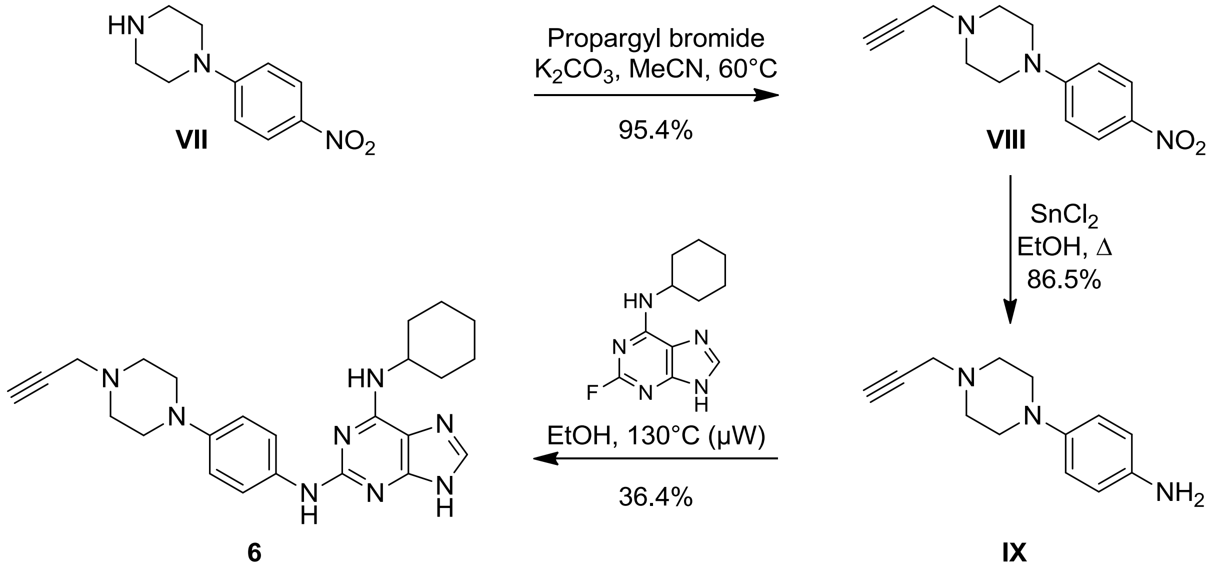

N-(4-nitrophenyl)-N'-propargylpiperazine (VIII)

To a suspension of *N*-(4-nitrophenyl)piperazine **VII** (1.0 g, 4.83 mmol) and K_2CO_3 (868 mg, 6.28 mmol) in MeCN (24 mL) was added propargyl bromide (651 µL, 80% in toluene, 6.04 mmol). The reaction mixture was heated to 60°C with vigorous stirring for 18 hours. After cooling to RT, the mixture was concentrated *in vacuo*. The residue was purified by silica gel chromatography (MeOH/CH_2Cl_2, 3:97 v/v) to yield the title compound (1.13 g, 4.61 mmol, 95.4%) as an amorphous yellow solid, which upon crystallization (CH_2Cl_2/hexane) gave fine needles. ^{1}H NMR (300 MHz, $CDCl_3$) δ = 8.12 (d, 2H, *J* = 9.6 Hz), 6.83 (d, 2H, *J* = 9.3 Hz), 3.47 (t, 4H, *J* = 5.3 Hz), 3.39 (d, 2H, *J* = 2.4 Hz), 2.72 (t, 4H, *J* = 5.3 Hz), 2.29 (t, 1H, *J* = 2.6 Hz); ^{13}C NMR (75 MHz, $CDCl_3$) δ = 154.8, 138.6, 126.0, 112.8, 78.1, 73.8, 51.3, 47.0, 46.8; HRMS: calcd. for $C_{13}H_{16}N_3O_2$ (M+H): 246.1237, found: 246.1250.

N-(4-aminophenyl)-N'-propargylpiperazine (IX)

Compound **VIII** (4.2 g, 17.3 mmol) and $SnCl_2$ (18.4 g, 96.8 mmol) were taken up in EtOH (62 mL). The resulting reaction mixture was refluxed at 70°C under vigorous stirring for 9 hours. After cooling to RT, the pH was made slightly basic (pH 7-8) by slow addition of sat. $NaHCO_3$. The alkaline solution was transferred to a separation funnel and the aqueous fraction was extracted thrice with EtOAc (60 mL). All EtOAc fractions were pooled, dried over Na_2SO_4 and taken to dryness. The residue was purified by silica gel chromatography (MeOH/CH_2Cl_2, 6:94 v/v) yielding the title compound (1.9 g, 8.9 mmol, 86.5%) as a beige amorphous solid. ^{1}H NMR (300 MHz, $CDCl_3$) δ = 6.80 (d, 2H, *J* = 8.7 Hz), 6.62 (d, 2H, *J* = 8.7 Hz), 3.41 (br s, 2H), 3.34 (d, 2H, *J* = 2.4 Hz), 3.08 (t, 4H, *J* = 5.0 Hz), 2.72

(t, 4H, *J* = 5.0 Hz), 2.28 (t, 1H, *J* = 2.3 Hz); ^{13}C NMR (75 MHz, $CDCl_3$) δ = 144.3, 140.2, 118.6, 116.1, 78.8, 73.4, 52.0, 50.8, 46.8; HRMS: calcd. for $C_{13}H_{18}N_3$ (M+H): 216.1495, found: 216.1489.

N^6-cyclohexyl-2-(4-(4-propargylpiperazin-1-yl)-*N*-anilinyl)-adenine (6)

N^6-cyclohexyl-2-fluoroadenine (235 mg, 1.0 mmol) and compound **IX** (431 mg, 2.0 mmol) were taken up in EtOH (3 mL) and the resulting reaction mixture was heated (µW) to 130°C for 5 hours. After cooling to RT, the mixture was transferred directly into a silica gel column (MeOH/CH_2Cl_2, 6:94 v/v). The thus obtained semi pure product was purified further by precipitation (CH_2Cl_2/hexane) yielding the title compound (157 mg, 0.36 mmol, 36.4%) as a beige amorphous solid. (More product could be isolated from the mother liquor, albeit in a significantly lower purity as the first crop.) ^{1}H NMR (300 MHz, $CDCl_3$) δ = 12.82 (br s, 1H), 7.42 (d, 2H, *J* = 8.7 Hz), 6.91 (d, 2H, *J* = 9.0 Hz), 6.74 (s, 1H), 6.65 (s, 1H), 5.56 (d, 1H, *J* = 6.3 Hz), 4.09 (m, 1H), 3.36 (d, 2H, *J* = 2.1 Hz), 3.18 (t, 4H, *J* = 4.8 Hz), 2.74 (t, 4H, *J* = 4.7 Hz), 2.28 (t, 1H, *J* = 2.1 Hz), 2.13-2.02 (m, 2H), 1.83-1.71 (m, 2H), 1.70-1.59 (m, 1H), 1.50-1.14 (m, 5H); ^{13}C NMR (75 MHz, $CDCl_3$) δ = 157.3, 154.5, 150.6, 147.9, 135.9, 132.6, 123.3, 117.5, 114.6, 78.8, 73.5, 52.0, 50.0, 49.3, 47.0, 33.4, 25.8, 25.1; HRMS: calcd. for $C_{24}H_{31}N_8$ (M+H): 431.2666, found: 431.2689.

Conjugate X

Azide **2a** (160 mg, 0.2 mmol) was taken up in a mixture of TFA and CH_2Cl_2 (5 mL, 1:1, v/v) and stirred for 40 minutes at RT. The reaction mixture was then taken to dryness, coevaporated twice with toluene and concentrated under high vacuum for 1 hour. The residue was taken up in a mixture of water and *tert*-butanol (3 mL, 1:1, v/v) and alkyne **6** (43 mg, 0.1 mmol), $CuSO_4$ (20 µL, 0.5M, 0.1 eq.) and Na-ascorbate (100 µL, 0.5M, 0.5 eq.) were added. Finally, the resulting reaction mixture was charged with a catalytic amount of TBTA[4] and Et_3N and heated to 80°C under vigorous stirring for 72 hours. After cooling to RT, the solution was concentrated *in vacuo* and the residue was purified by preparative RP-HPLC (MeCN/water/formic acid 10 → 100%) yielding the title compound (36 mg, 31 µmol, 31.0%) as a pale yellow amorphous solid. LC-MS: Rt = 5.17 min (10 → 100% MeCN, 15 min run); HRMS: calcd. for $C_{56}H_{79}N_{20}O_9$ (M+3H): 391.8774, found: 391.7788.

NMR Assignment of the "left" part of conjugate X

The labelling of the molecule's common part is depicted in the following figures:

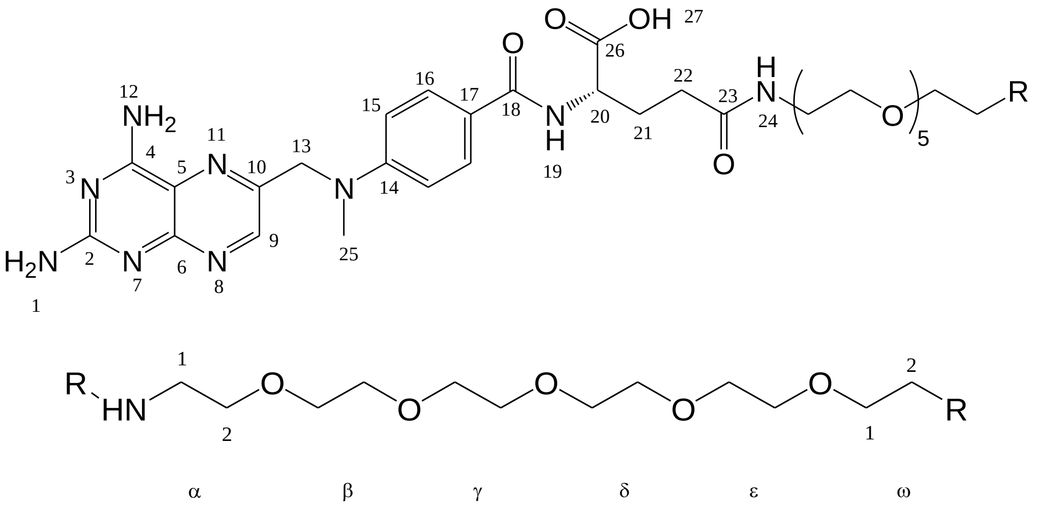

Figure 5: Labeling scheme of common part of MFCs **X** and **4a** and their PEG tail

Nearly complete assignment of the ^{1}H and ^{13}C resonances visible in the spectra could be achieved (see Table 5).

Since there is only one CH_3 group in molecule **X**, Me-25 can, with its integration value, easily be assigned to the singlet at 3.19 ppm. Starting from this singlet there are two HMBC correlations, one to a CH_2 at 54.8 ppm and one to a C_q at 150.6 ppm, which is C-14. The CH_2 signal has a proton resonance at 4.76 ppm and is a singlet, thus it can be assigned to be H-13. It also shows a COSY correlation to both Me-25 and a signal in the aromatic region which can be assigned to H-9. Also, H-13 shows an HMBC correlation to a C_q at 145.9 ppm, this is C-10. There is also a HMBC correlation visible from H-9 to a C_q, which is thus tentatively assigned to C-6.

From Me-25 and H-13 there is a COSY correlation to an aromatic triplet (in **X**) at 6.83 ppm, this however is a pseudo triplet from two doublets that are overlapping. The left doublet can be assigned to the two H-15 protons. For DC010, there is no overlap and the doublet at 6.83 ppm is well resolved. From this resonance, a COSY correlation to a doublet at 7.71 ppm is visible, this is H-16. H-15 has an HMBC correlation to 121.1 ppm, which is assigned to C-17, and H-16 has an HMBC correlation to 150.6 ppm which was already assigned as C-14. H-16 additionally has a small HMBC to a C_q at 166.0 ppm that is tentatively assigned to C-18. From H-16 there is a NOESY correlation to a broad resonance at 8.18 ppm that does not have an HSQC correlation and can therefore be assigned to the H-19 amine. From this position, a COSY correlation is visible to a resonance at 4.25 ppm, which is according to the HSQC a CH and can be assigned to H-20. From H-20 there are two COSY correlations to 2.04 and 1.9 ppm, looking at the integrations and the HSQC spectrum, the one at 2.04 and the right part of 1.9 ppm together can be assigned to the two protons H-21 (for **4a** the multiplet at 1.9 ppm is not overlapping with another signal). From these resonances, a COSY correlation is visible towards 2.182 ppm, with HSQC and the integral value; this is assigned to be H-22. Both resonances from H-21 and H-22 have a NOESY correlation to the amine H-19. From H-22 there is also a NOESY correlation to a triplet at 7.88 ppm which has an integral of 1 and no HSQC

correlation and can be assigned to the H-24 amine. From H-24 a COSY correlation to a quadruplet at 3.16 ppm is visible that has an HSQC correlation to a CH_2 and an integral value of two and can be assigned to H-α1. From this one, a COSY correlation to a triplet at 3.35 ppm with an HSQC correlation to a CH_2 is visible, that can be assigned to H-α2. From H-24, H-21 and H-α1 an HMBC correlation is visible towards a C_q at 171.81 ppm, that can be assigned to C-23. From H-α2, HMBC correlations to 69.6 ppm are visible; these correlate to multiple CH_2's between 3.4 and 3.55 ppm and are thus assigned to the CH_2's β to δ. Another resonance at 3.82 ppm (triplet) also shows an HMBC to these CH_2 carbons; this resonance is a CH_2 and can be assigned to H-ω1. And from this one, there is a COSY correlation to a triplet at 4.5 ppm that has a HSQC correlation to a CH_2, which is H-ω2.

The H-1 and H-12 amines are present in the region 6.5-8 ppm; this is in agreement with the integrations, they can however not be distinguished. $O_{27}H$ is assigned at 12.29 ppm. In the most concentrated sample, DC010, a small HMBC correlation is visible from H-20 and H-21 to a quaternary carbon at 173.7 ppm; this is assigned to C-26.

Table 5: *Assignment of MTX-R in dmso-d6 at 298K*

Label	^{1}H (ppm)	^{13}C (ppm)	Multiplicity
1 + 12	n.a.	-	
2	-	n.a.	
4	-	n.a.	
5	-	n.a.	
6	-	155.21*	
9	8.55	148.9	s
10	-	145.9	
13	4.76	54.54	s
14	-	150.8	
15	6.82	110.72	d (pseudo t)
16	7.71	128.53	d
17	-	121.2*	
18	-	166.0*	
19	8.18	-	s
20	4.25	52.11	q
21	2.04+1.90	26.41	m
22	2.18	31.7	m
23	-	171.7	
24	7.88	-	t
25	3.19	38.83	s
26	-	173.7	
27	12.29	-	
α1	3.16	38.21	q
α2	3.35	68.76	t
β–δ	3.4-3.55	69.6	
ω1	3.82	68.5	t
ω2	4.50	48.9	t

* tentative assignment only

NMR Assignment of the distinct part of conjugate X

The labelling scheme of the distinct part of **X** is depicted below.

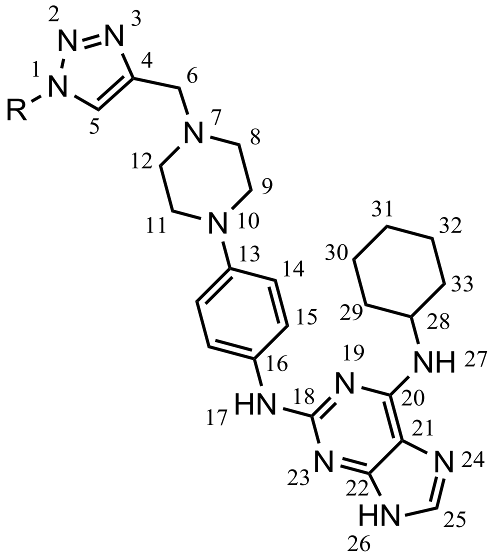

Figure 6: Labeling scheme of the right part of DC009

From the ω2 CH_2 protons in the PEG chain, there is an HMBC correlation to a CH at 124.1 ppm. With HSQC this is seen to correlate to a ^{1}H singlet at 7.98 ppm, this is H-r5. Another HMBC correlation to C-r5 comes from a CH_2 singlet at 3.60 ppm, this is H-r6. Starting from H-r6, two more HMBC correlations are seen, one to a C_q at 143.4 ppm which is assigned to be C-r4 and one to a CH_2 at 52.14 ppm that has an HSQC correlation to 2.54 ppm and integrates for 4 protons, and can be assigned to the isochronous H-r8 and H-r12. From this position there is a COSY correlation to a signal at 3.01 ppm that integrates for 4 protons and can be assigned to the isochronous H-r9 and H-r11. From this last resonance, a NOESY correlation to both 6.8 ppm and 7.63 ppm is visible. The one at 6.8 ppm is a pseudo triplet, which is actually consisting out of two CH doublets that both integrated for 2 protons. The doublet on the right can be assigned to H-r14. The other doublet at 7.64 ppm (on the left) can be assigned to H-r15. An HMBC correlation is visible from H-r14 to a C_q at 134.4 ppm that is assigned to C-r16. Also from H-r15 an HMBC correlation is visible to a C_q at 145.1 ppm that is assigned to C-r13. A NOESY correlation is visible from H-r15 to a NH at 8.5 ppm that is assigned to the H-r17 amine.

Because of the large amount of nitrogen atoms and quaternary carbons, assignment from this point onwards was difficult. A TOCSY correlation was found between 5 CH_2 resonances that integrated together for 10 protons and a CH resonance at 4.05 ppm, which is assigned to H-r28 (the resonance is however broadened and no HSQC correlation was found). Distinction between the 5 CH_2 signals to assign them to r29-33 is difficult and only done tentatively. A NOESY correlation is visible from H-r28 and the CH_2 at 1.33 ppm to a NH signal at 7.04 ppm that is assigned to the H-r27 amine.

H-r25 is only assigned tentatively by means of exclusion, there is only one singlet left in the aromatic region and this is therefore very likely to be H-r25. The remaining quaternary carbons C-22, C-21, C-20 and C-18 are not

assigned due to lack of HMBC correlations. Also the amine H-26 is not assigned. Nevertheless, the integrity of the chemical link between the PEG chain and the clicked moiety could be established without ambiguity.

Table 6: *Assignment of **X** in dmso-d6 at 298K*

Label (r)	^{1}H (ppm)	^{13}C (ppm)	Multiplicity
4	-	143.1	
5	7.98	123.7	s
6	3.60	52.34	s
8+12	2.54	52.11	br t
9+11	3.01	49.1	br t
13	-	145.1	
14	6.80	115.7	d
15	7.63	119.0	d
16	-	134.4	
17	8.48	-	br s
18	-	n.a.	
20	-	n.a.	
21	-	n.a.	
22	-	n.a.	
25	8.16*	n.a.	
26		-	
27	7.04	-	
28	4.05	?	
29	1.33*	24.9	
30	1.33*	24.9	
31	1.15+1.63*	25.0	
32	1.76*	24.9	
33	1.33+1.93*	32.3	

* tentative assignment only

Synthesis of the FK506 MFC

4-(trimethylsilylethynyl)phenol (XI)

To a solution of 4-iodophenol (2.2 g, 10 mmol) in triethylamine (30 mL) were added $Pd(PPh_3)Cl_2$ (70 mg, 0.1 mmol, 1 mol%), CuI (19 mg, 0.1 mmol, 1 mol%) and trimethylsilylacetylene (2.8 mL, 20 mmol) and the solution was heated to 80°C under vigorous stirring. After 6 h, the reaction mixture was cooled to RT, filtered and concentrated *in vacuo*. Chromatography of the dark colored residue on silica gel ($MeOH/CH_2Cl_2$, 1:99 v/v) yielded the product as a light brown solid (1.83 g, 9.6 mmol, 96%). Recrystallization from heptanes yielded the product as off white needles. ^1H NMR (300 MHz, $CDCl_3$) δ = 7.35 (d, 2H, *J* = 8.7 Hz), 6.74 (d, 2H, J = 8.7 Hz), 5.13 (s, 1H), 0.23 (s, 9H); ^{13}C NMR (75 MHz, $CDCl_3$) δ = 155.7, 133.7, 115.5, 115.3, 105.1, 92.6, 0.1; HRMS calcd. for $C_{11}H_{13}OSi$ (M-H): 189.0741, found: 189.0733.

Compound 9

Alcohol **7**[viii] (104 mg, 0.1 mmol) and p-(TMS-ethynyl)phenol (**XI**) (38 mg, 0.2 mmol) were placed in a flask under a nitrogen atmosphere. To the dry material was added a solution of PPh_3 in toluene (1.3 ml, 0.1M, 1.3 eq.). The solution was cooled on ice and dropwise a solution of DEAD in toluene (1.3 mL, 0.1M, 1.3 eq.) was added and the solution was stirred overnight. The reaction mixture was transferred directly into a silica gel column and the product (106 mg, 87.7 µmol, 87.7%) was obtained after elution (0→25% EtOAc in toluene). HRMS calcd. for $C_{66}H_{113}N_2O_{13}Si_3$ ($M+NH_4$): 1225.7545, found: 1225.7505. The product is taken up in MeCN (4 mL), cooled on ice. To the solution are added acetic acid (30 µl, 526 µmol, 6 eq.) and TBAF (525 µl, 1.0M in THF, 525 µmol, 6 eq.) and the mixture was stirred overnight. The solution was cooled on ice again and HF/pyridine (100 µl) was added and stirring was continued for 6 h. The solution was poured into sat. $NaHCO_3$ (20 mL) and transferred to a separation funnel. The aqueous fraction was extracted with EtOAc (4 x 25 mL) and the organic fractions were pooled, dried (Na_2SO_4) and concentrated. The residue was purified in silica gel (0→10% MeOH in CH_2Cl_2) to yield the product (73 mg, 80.4 µmol, 80.4% based on **7**) as an off white foam. ^1H NMR (700 MHz, DMSO-d_6) (mixture of rotamers) δ = 7.38 (2H, d); 6.87 (2H, d), 6.54 (0.5H, s), 5.25 (0.5H, d); 5.11 (1H, m); 5.07 (0.5H, d); 5.04 (0.5H, dd), 4.80 (1.5H, br m); 4.70 (0.5H, d); 4.63 (1H, t); 4.42 (0.5H, m); 4.21 (0.5H, d); 4.00 (1H, d), 3.93 – 3.78 (3H, m); 3.74 (0.5H, dt); 3.68 (0.50H, m); 3.57 – 3.36 (2.5H, m); 3.34 – 3.13 (15H, mm); 2.91 (1.50H, m); 2.74 (0.5H, m); 2.62 (0.5H, m); 2.44 (0.5H, m), 2.36 (0.50H, dd), 2.29 – 0.9 (36.5H, mm), 0.9 – 0.7 (9H, m), 0.62 (1.5H, d); HRMS calcd. for $C_{51}H_{73}N_NO_{13}Na$ (M+Na): 930.4974, found: 930.5010.

NMR analysis of the FK506 derivative 9

The spectra are quite complex, and a full assignment has not been attempted. Given that the synthesis of **9** starts out from commercially available FK506. A lot of resonances are apparent. The labeling scheme for **9** is presented hereafter.

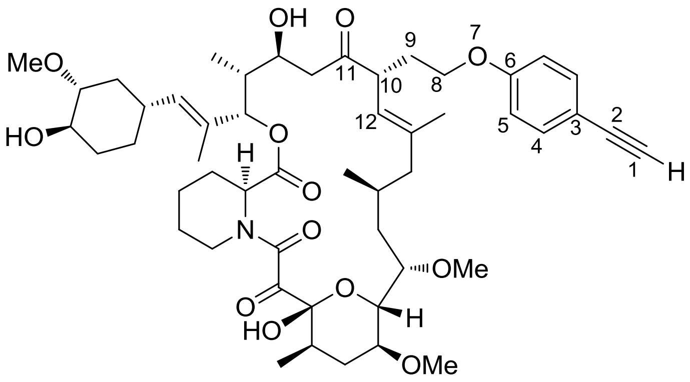

Figure 7: Labelling scheme for relevant part of **9**

Analysis starts by noting the alkyne proton to carbon correlations in the HSQC, which have a typical position and appearance due to the unusual $^{1}J_{CH}$ (~250 Hz) and $^{2}J_{CH}$ (~50 Hz) scalar coupling constant. As a result of the large size of the latter, a cross-peak is visible in the HSQC spectrum representing a –***C**$_q$*≡C–***H*** correlation. The corresponding carbons are at 83.79 (C_q) and 79.17 (C–H) ppm. Their identity is further confirmed by noting the residual ^{1}J scalar coupling doublet artefacts that occur at the frequency positions of the alkyne Cq. Because it is correlated to the alkyne proton via $^{2}JC_qH$ coupling of 51.4 Hz, the ^{2}JCH splitting is not removed by the low-pass filter set at 140 Hz. Thus both the chemical shift and the appearance confirm the assignment to the alkyne functionality in **9**.

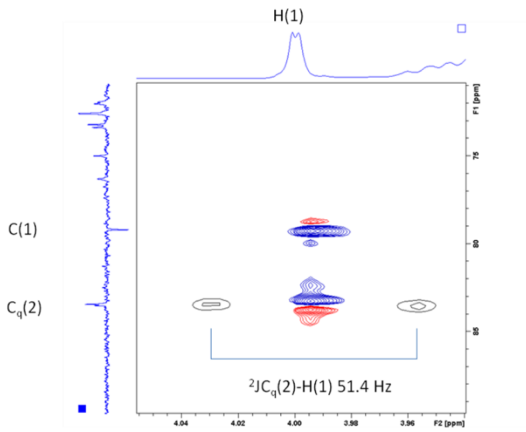

Figure 8. Superposition of a detail of the ^{1}H-{^{13}C} HSQC spectrum (red/blue for -/+ intensity) with the ^{1}H-{^{13}C} HMBC spectrum (black) showing the residual ^{2}JCH splitting artefact at the level of the Cq(2) carbon. The antiphase nature of the correlation in the HSQC results from the large mismatch between the set and actual $^{n}J_{CH}$ couplings.

Continuing from the alkyne proton unit, an additional ^{n}JCH correlation involving a Cq and CH carbon at 113.61 respectively 133.19 ppm can be identified. These correspond to carbon 3 and 4 of the benzene-like moiety

introduced on the FK506 basic structure. The associated proton shift of H4 is 7.38 ppm, and features a triplet multiplicity. The latter at first appears odd, since only a doublet is expected. A strong COSY correlation is visible to the aromatic proton centered at 6.88 ppm which therefore corresponds to proton 5, the second proton of the aromatic cycle. The latter signal shows a double doublet like structure, which is also not expected (Figure 2, left).

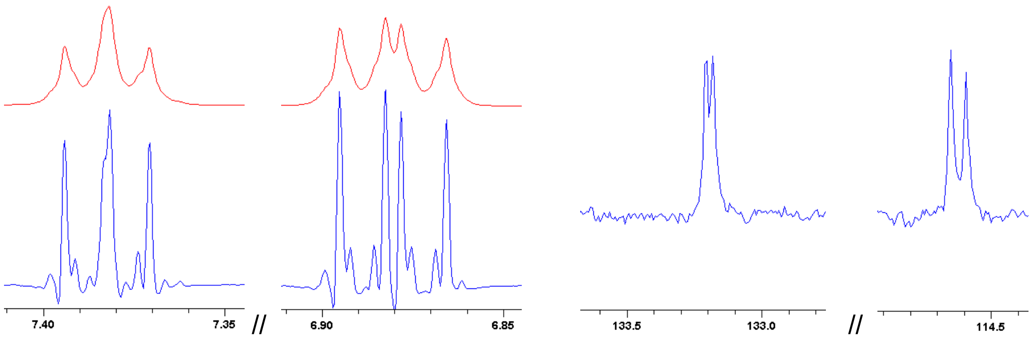

Figure 9: details of the ^{1}H (left) and ^{13}C APT (right) spectra showing the proton and carbon resonances corresponding to positions 4 and 5 in **12**. In the 1H spectrum, the bottom spectra represent a resolution enhanced version of the top spectra, obtained through Gaussian window multiplication.

Gaussian resolution enhancement of the 1D proton spectrum shows the presence of two equally intense doublets with overlapping lines, causing the apparent triplet in the regularly processed 1D proton spectrum. Also, the ^{13}C APT shows that the carbon resonances associated with H4 and H5 each consist of two equally intense CH type resonances. Because the lines are equally intense, one could conclude that the proton and carbon resonances at position 4 and 5 of **12** are non-equivalent, for instance as a result of hindered rotation which results in slow or no exchange on the NMR time scale. However, the carbon at C6 also appears as two equally intense resonances (*vide infra*) ruling out the possibility of slow rotation around the C6-O7 bond as the origin of the non-equivalence. We noticed that splitting of the expected resonances in two equally intense ones, both in the ^{1}H and ^{13}C spectrum, occurred frequently and that the chemical shift separation between both increased as one progressed towards the macrocyle. Based upon literature reports describing the assignment of FK506, we propose that this phenomenon is explained by the presence of cis-trans isomers around the tertiary amide bond, causing two stereoisomers in slow or no exchange.[ix] For FK506 in $CDCl_3$ f.i. the cis-trans ratio comprises 2:1. FK506 was reported as insoluble in dmso-d6, thus there is no information on the expected cis-trans ratio in dmso. It would appear that the modification to the FK506 structure in **12** results in solubility in dmso-d6 and a 1:1 ratio of both stereoisomers. However, this needs confirmation via a complete conformational analysis of 12, which was not attempted here. Unless otherwise mentioned, chemical shifts reported hereafter generally correspond to the midpoint or only one of the closely spaced resonances in the proton and carbon spectra.

Both H4 and H5, are connected to a quaternary carbon at 158.85 ppm which represent the Cq at the foot of the alkoxy fragment, i.e. position 6. In the NOESY spectrum H5 shows strong nOe correlations to protons at 3.95 and 3.85 ppm. These two protons correlate to a single CH_2 type carbon at 65.37 ppm in the multiplicity edited HSQC,

identifying the CH_2 at position 8. This assignment is confirmed by long range ^{n}JCH correlations to Cq(6) at 158.85 ppm.

The area of the proton spectrum in the vicinity of the $–CH_2–O$ at position 8 is heavily overlapped, complicating analysis. The TOCSY suggests additional correlations to four other protons at 4.71, 3.69, 2.08 and 1.71 ppm respectively. Cross-peaks connect the CH2 at position 8 directly with the resonances at 2.08 and 1.71, identifying the latter as the CH_2 unit at position 9, with its carbon at 30.26 ppm. This carbon also correlates to the protons at position 8. Both CH_2's (8 and 9) are found to correlate in the HMBC to two CH units located at 49.19/3.69 and 49.29/3.73 respectively. Again, both protons together integrate for a single 1H compared to the aromatic signals, indicating that these should be considered as the same proton at position 10, but in separate isomers. This is conformed from the TOCSY and COSY. Finally, the –C(9)H2-C(10)H- fragment shows ^{n}JCH to the keto-carbonylregion, with two peaks at 209.3 and 210.24 ppm respectively, corresponding to C11. With this, the macrocycle of the FK506 structure has been reached.

In the HSQC, 4 CH peaks connecting to the sp^2 carbon region can be seen at (5.11;131.72); 5.10;130.24); (4.80;122.17) and (4.70;121.32) respectively. The latter group shows clear correlations to the previously assigned spin system. The other CH proton appears to connect to a very extended (>10 protons) spinsystem in the TOCSY, corresponding to the branch of the macrocycle ring extending in the direction of C12 and beyond.

Based upon the analysis, the identity of the modification to the FK506 basic structure could be unambiguously determined, supporting the structure of **9** as presented.

Table 7: Partial NMR a*ssignment of **9** in DMSO-d$_6$ at 298K (700/176 MHz) according to scheme above.*

Label	^{1}H (ppm)	^{13}C (ppm)	Multiplicity
1	4.00	79.17	d 1.83 Hz
2	-	83.79	
3	-	113.61	
4_1	7.39	133.21	d 8.8 Hz
4_2	7.38	133.18	d 8.8 Hz
5_1	6.89	114.65	d 8.8 Hz
5_2	6.87	114.59	d 8.8 Hz
6_1	-	158.89	
6_2	-	158.82	
8	3.93/3.84	65.37	m/m
9	2.08/1.71	30.26	m/m
10_1	3.73	49.29	dt 10.14/7.16 Hz
10_2	3.69	49.19	ddd 9.8/8.2/5.4 Hz
11_1	-	210.24	
11_2		209.30	
12_1	4.80	122.17	d 10.1
12_2	4.70	121.32	m (overlapped)

* the subscripts 1 and 2 refer to the two different rotamers present in solution. The label was attributed based upon the relative position of the peaks (highest ppm first), not by assignment to a specific rotamer.

Conjugate 10

Azide **2a** (47 mg, 63 µmol) is taken up in a mixture of TFA and CH_2Cl_2 (1.5 mL, 1:1, v/v), stirred for 40 minutes at RT, taken to dryness and coevaporated twice with toluene. The residue is taken up in a mixture of water and *tert*-butanol (1 mL, 1:1, v/v) and alkyne **9** (30 mg, 33 µmol), $CuSO_4$ (35µl, 1.0M, 1.0 eq.) and ascorbic acid (165 µL, 1.0M, 5.0 eq.) are added. The reaction is stirred overnight at room temperature. The solution is concentrated *in vacuo* and purified with preparative RP-HPLC (MeCN/water/formic acid 10→90%). HRMS calcd. for $C_{83}H_{120}N_{13}O_{22}$ (M+H): 1650.8665, found: 1650.9032.

2 Molecular biology: MASPIT assay, experimental procedures and results

The pCLL-eDHFR receptor-DHFR vector was cloned by transferring the *E. coli* DHFR insert from pSEL-eDHFR[x] into the pCLL backbone[xi] using SacI and NotI restriction sites. The pCLG-eDHFR receptor-DHFR plasmid was similarly produced by transferring the *E.* coli DHFR insert into the pCLG backbone.[xii] The empty prey construct pMG2 has been previously described.[xiii] The FKBP12 prey construct pMG2-FKBP12 was generated by amplifying the FKBP12 coding sequence and cloning it into the EcoRI and NotI restriction sites of pMG2.[xiv] The prey plasmids pMG1-EFHA1, pMG1-ESR1 and pMG1-TTK were created by Gateway transfer of the full size EFHA1 ORF, obtained as an entry clone in the hORFeome collection,[xv] into the Gateway compatible pMG1 prey destination vector as described earlier.[13]

Cells were transfected using a standard calcium phosphate protocol as previously described.[13] In binary MASPIT assays, HEK293T cells were seeded in black tissue-culture treated 96-well plates at 10.000 cells/well in 100µl culture medium (DMEM supplemented with 10% foetal calf serum), and grown at 37°C, 8% CO_2. Twenty-four hours later, cells were transfected with combinations of receptor-DHFR and prey constructs and the pXP2d2-rPAP1-luciferase reporter. Twenty-four hours after transfection, cells were either left unstimulated or treated with 100ng/ml leptin, with or without addition of MTX-FK506 fusion compound. Another 24h later, luciferase activity was assayed using the Luciferase Assay System kit (Promega).

Microtiterplates (384well) for array screening, containing dried mixtures of prey and pXP2d2-rPAP1-luciferase reporter plasmids together with transfection reagent and additional components, were prepared as described, providing quadruplicate wells for each prey.[13] The collection used in this report contained a subset of 1879 full-length human ORF preys selected from the human ORFeome collection (Full list available in Supporting Information Table 1 of reference 11). To screen the plates, HEK293T cells were first seeded in T175 flasks at a density of 7E6 cells/flask in 35ml culture medium. After 24h, cells were transfected with the pCLL-eDHFR plasmid. Twenty-four hours after transfection, cells were detached and added to the array screening plates at 5.000 cells/well in 15µl medium. After 24h, duplicate wells were supplemented with 15µl MTX-FK506 containing medium (final concentration 1µM) or with 15µl medium containing MTX-FK506 and leptin (final concentration 1µM and 100ng/ml, respectively). Non-regiomeric MTX-FK506 conjugate was used in this experiment. Twenty-four hours after stimulation, luciferase activity was measured. Average values of the duplicate wells were normalized for the plate median value. Filters for data cleanup and to remove aspecifically binding preys were set as earlier described.[13]

3 Selected NMR spectra

Cmpd **2a** ^{1}H (700 MHz, DMSO-d6)

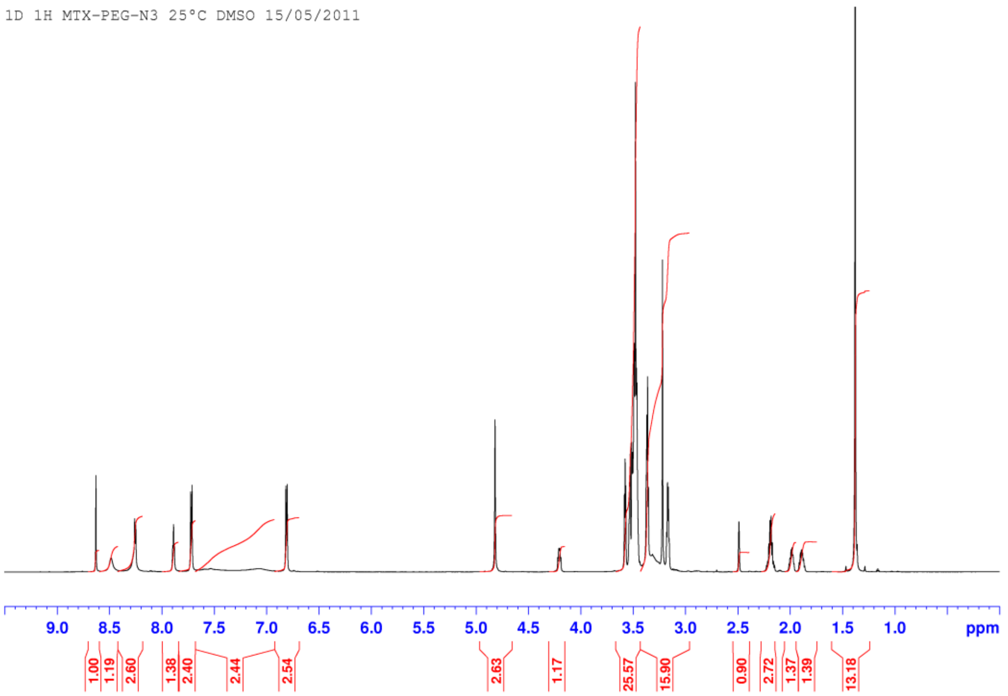

Cmpd **2a** ^{13}C APT (176 MHz, DMSO-d6)

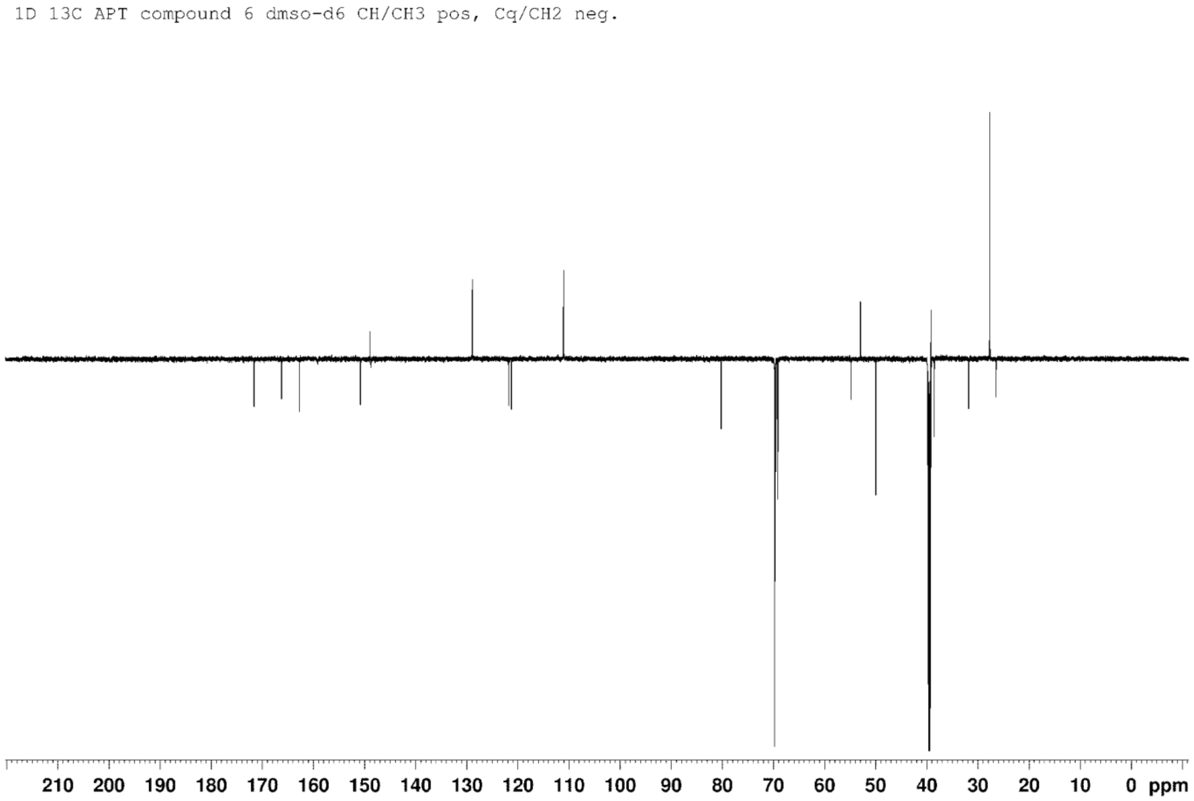

Cmpd **2a** ^{1}H-^{13}C geHSQC (700 MHz, DMSO-d6)

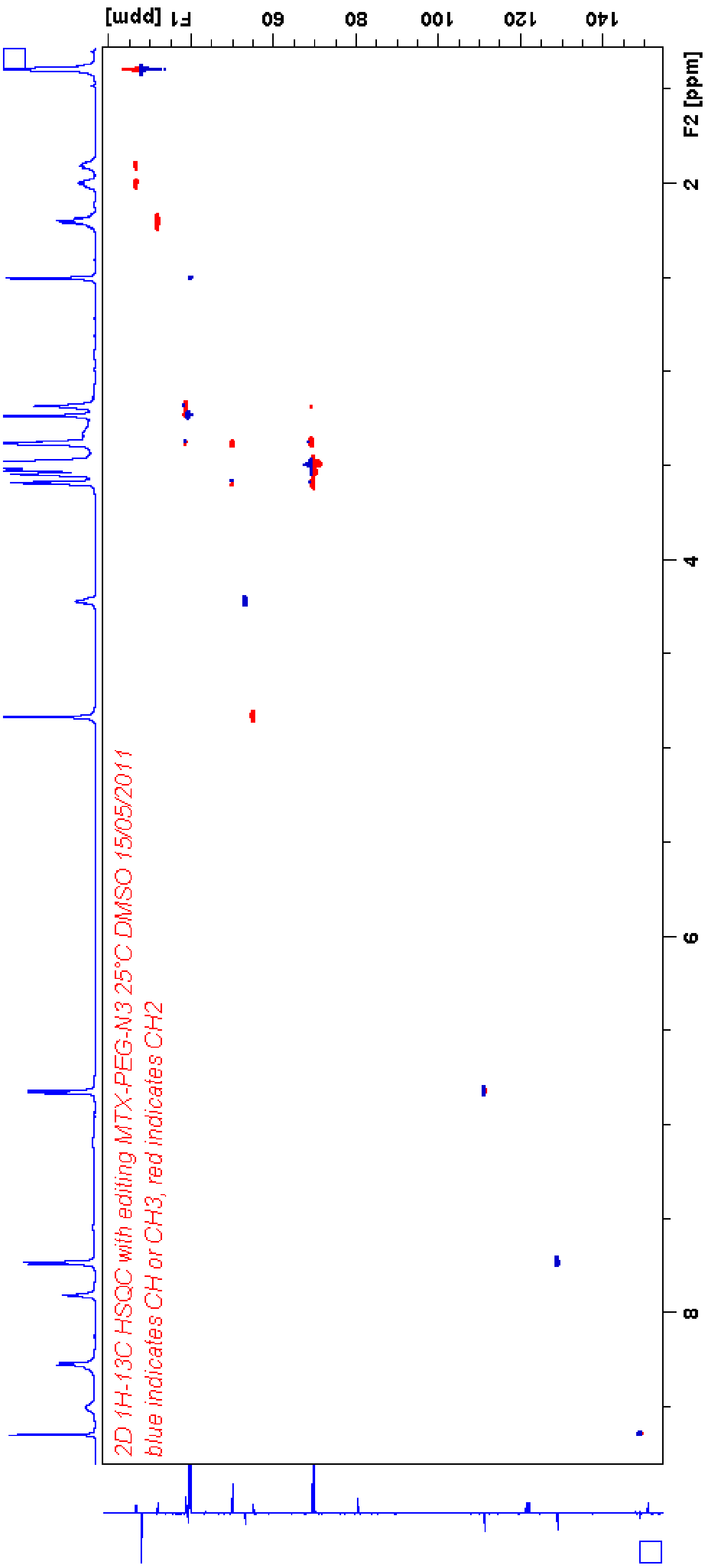

Cmpd **4a** ^{1}H (700 MHz, DMSO-d6)

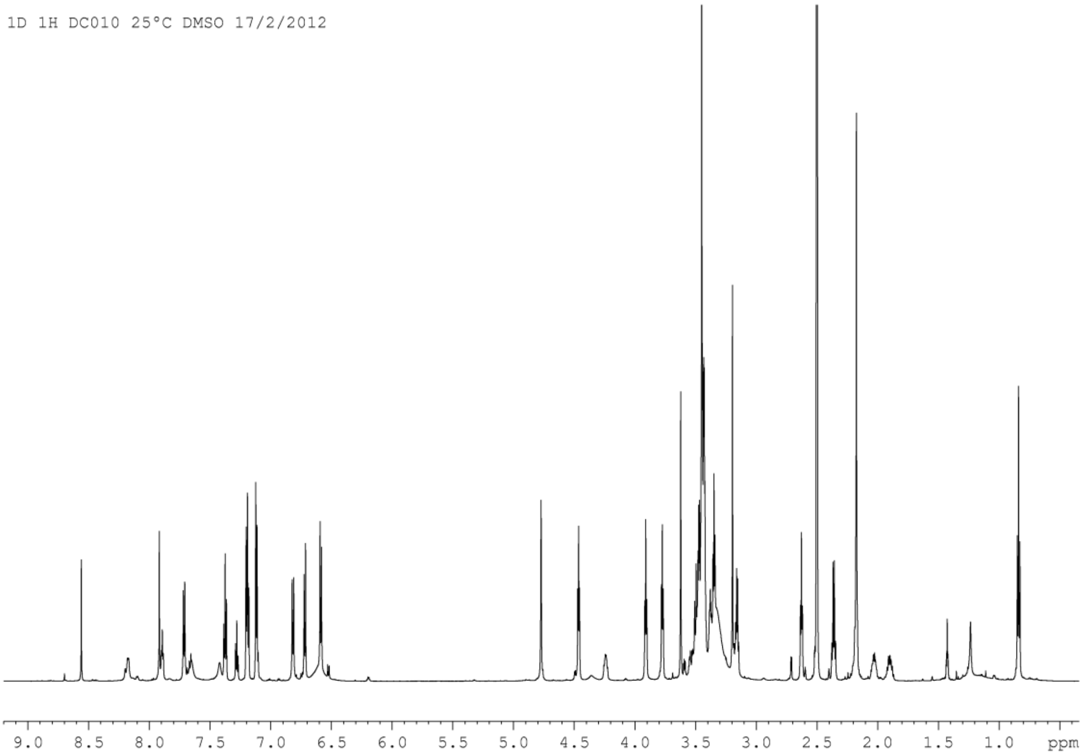

Cmpd **4a** ^{1}H-^{1}H COSY (700 MHz, DMSO-d6)

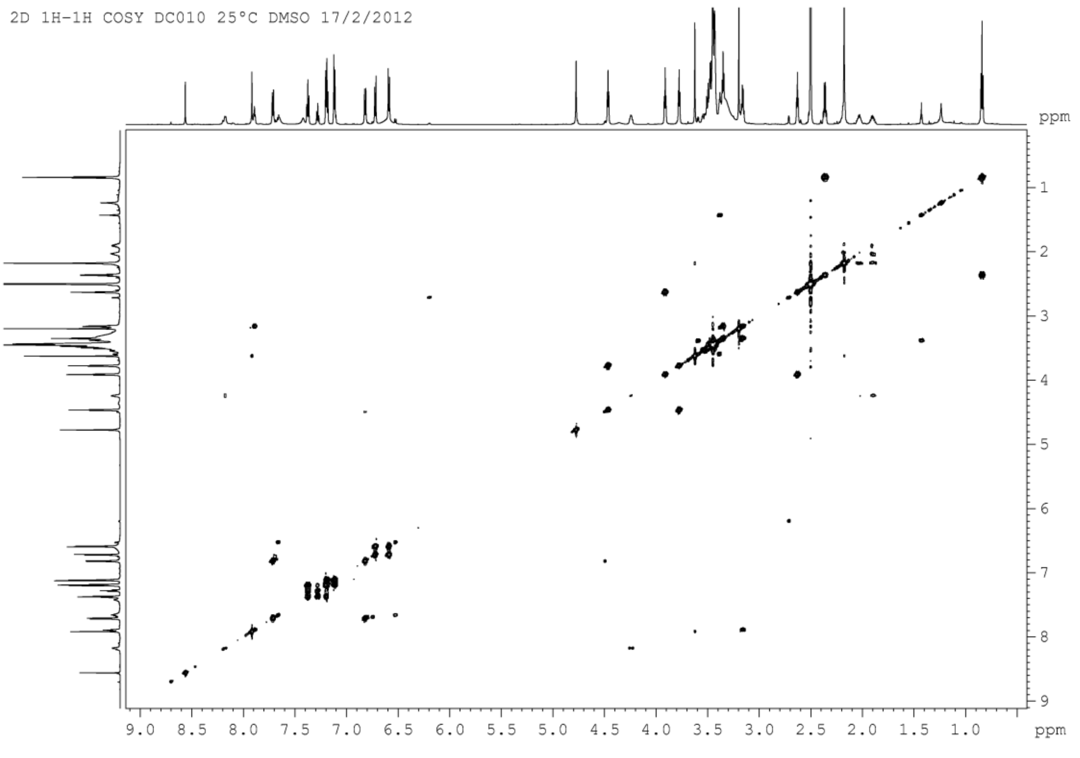

Cmpd **4a** ^{1}H-1H NOESY (700 MHz, DMSO-d6)

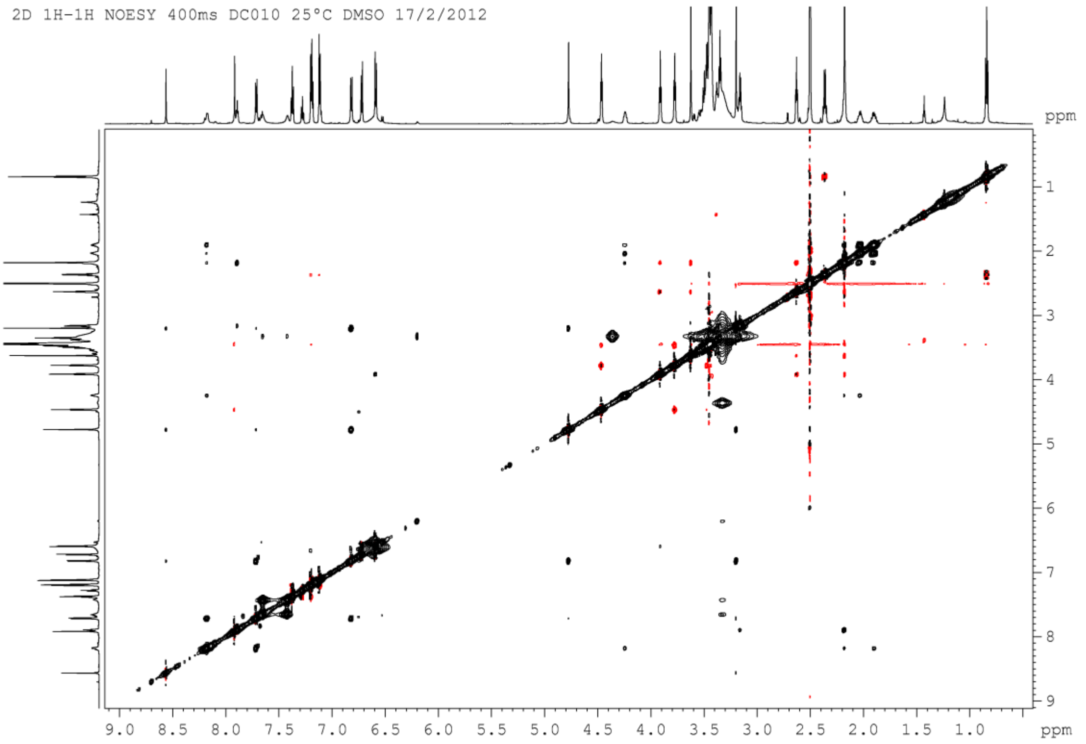

Cmpd **4a** ^{1}H-^{13}C geHSQC (700 MHz, DMSO-d6)

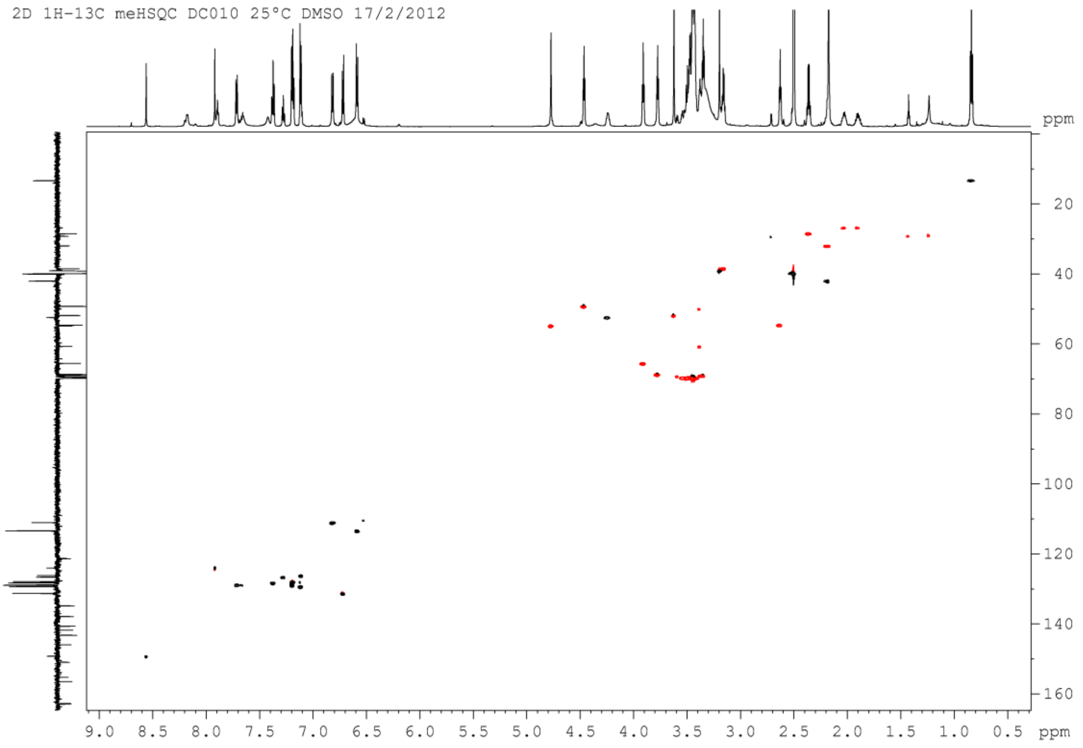

Cmpd **4a** ^{1}H-^{13}C geHMBC (700 MHz, DMSO-d6)

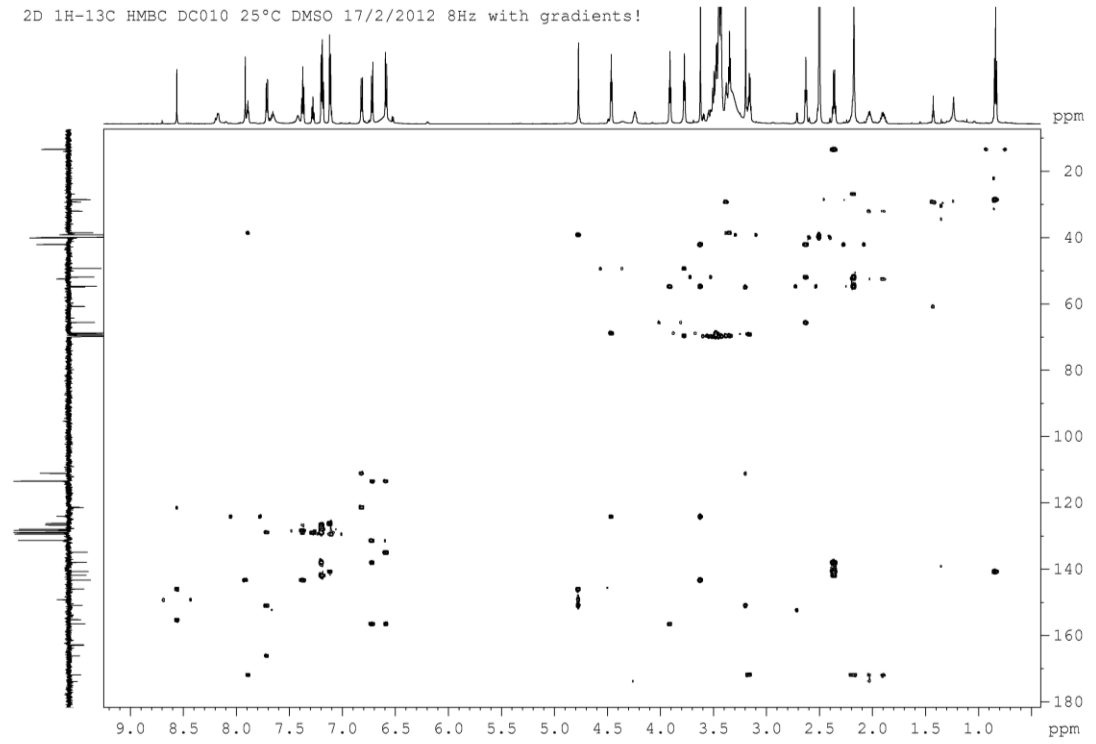

Cmpd **4a** ^{13}C APT (176 MHz, DMSO-d6)

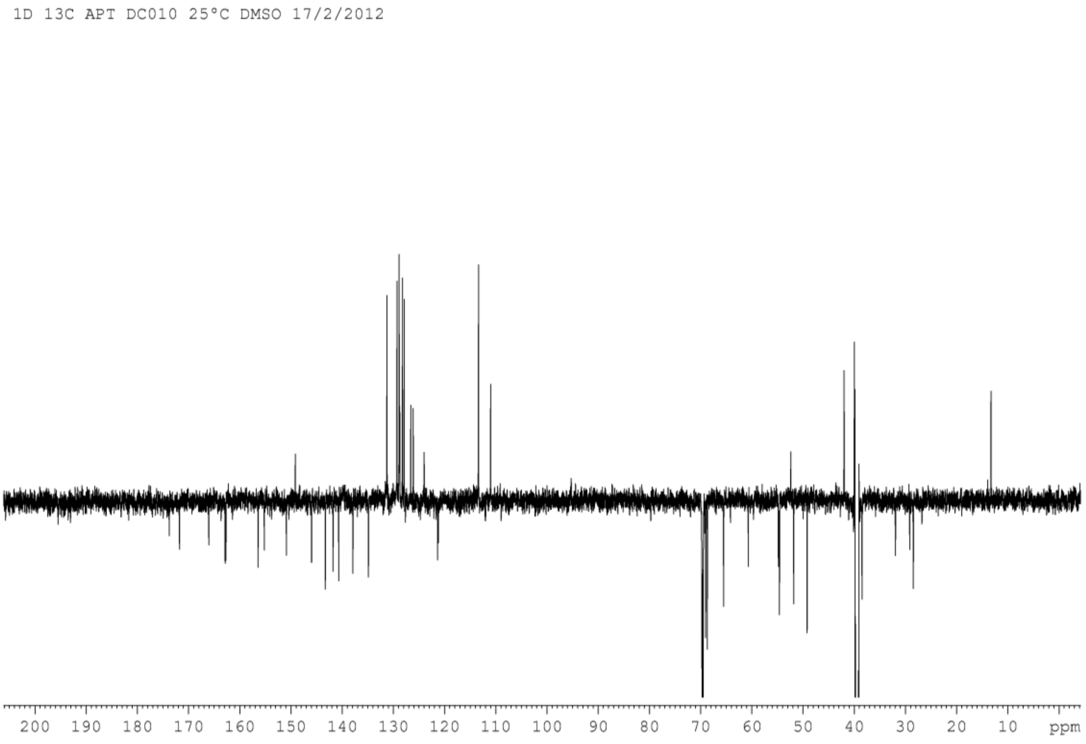

Cmpd **4a** TOCSY (700 MHz, DMSO-d6)

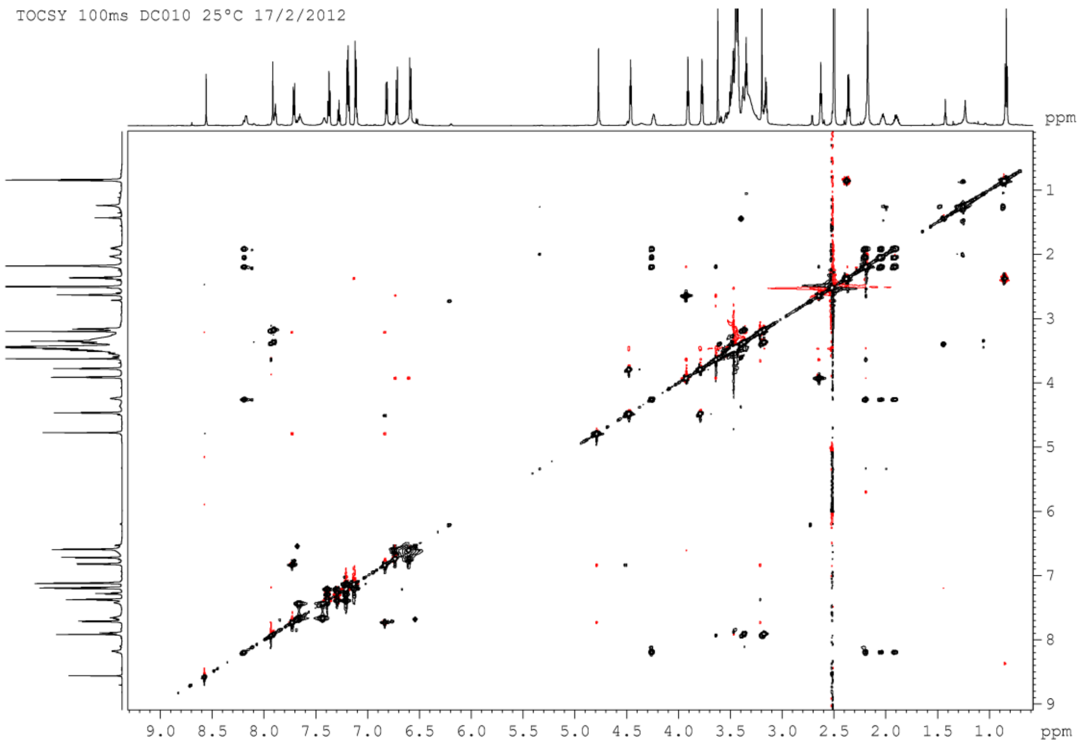

Cmpd **5** ^{1}H (700 MHz, DMSO-d6)

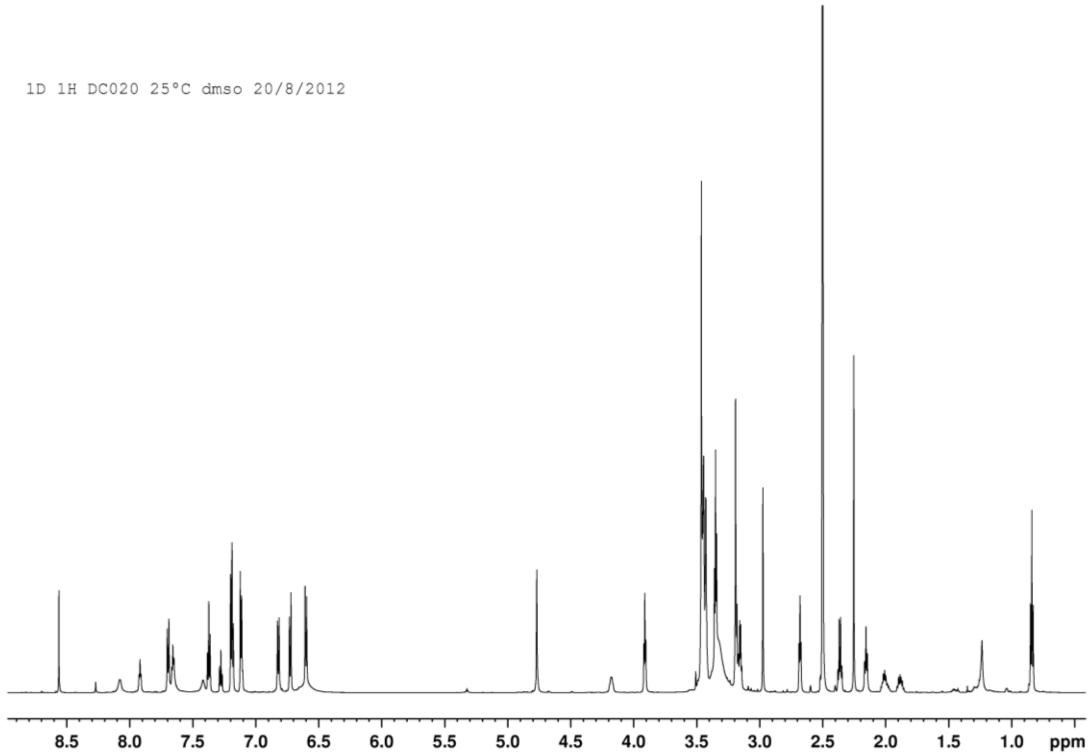

Cmpd **5** ^{1}H-^{1}H COSY (700 MHz, DMSO-d6)

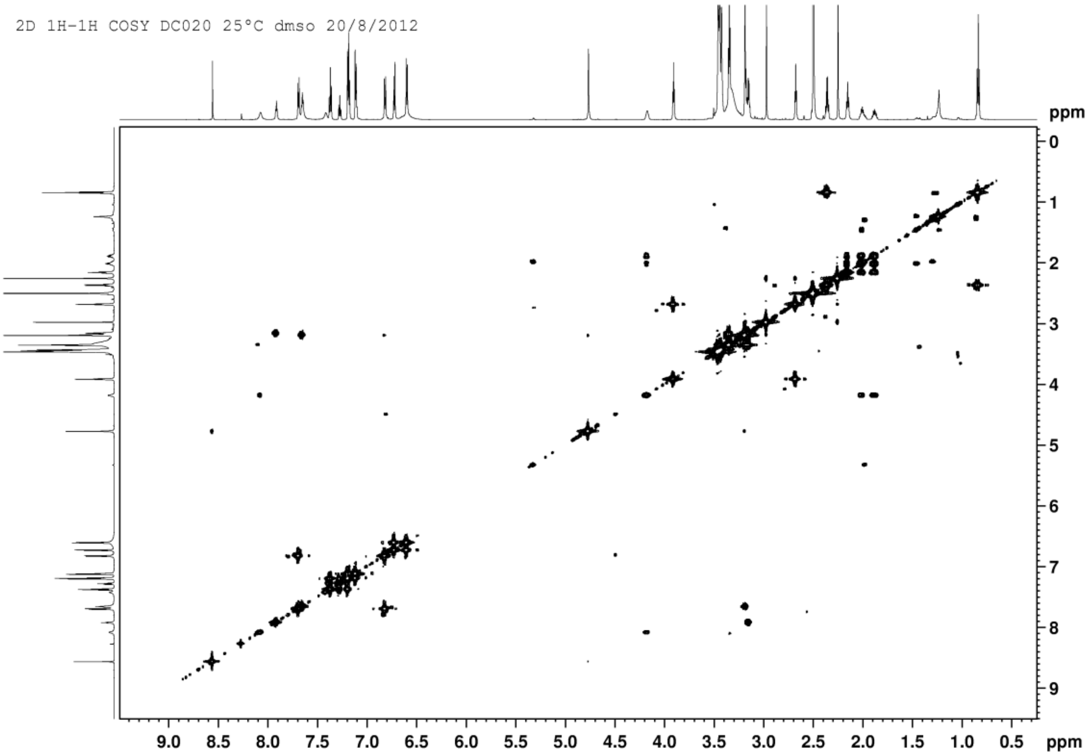

Cmpd **5** ^{1}H-1H NOESY (700 MHz, DMSO-d6)

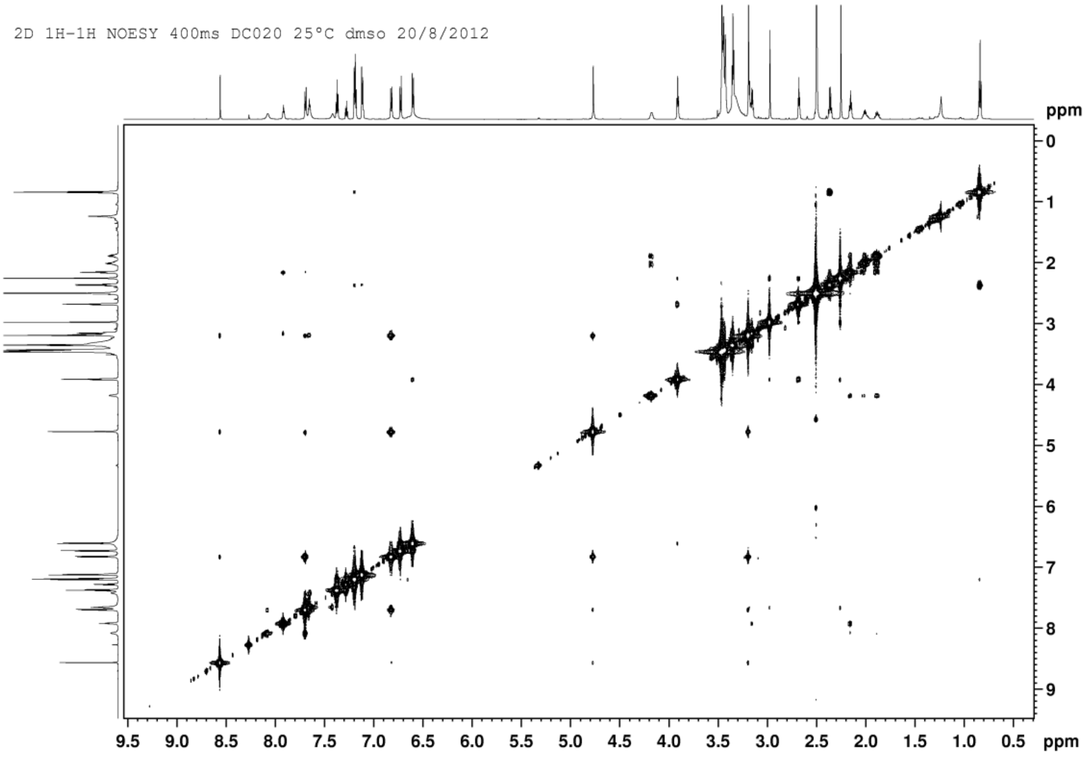

Cmpd **5** TOCSY (700 MHz, DMSO-d6)

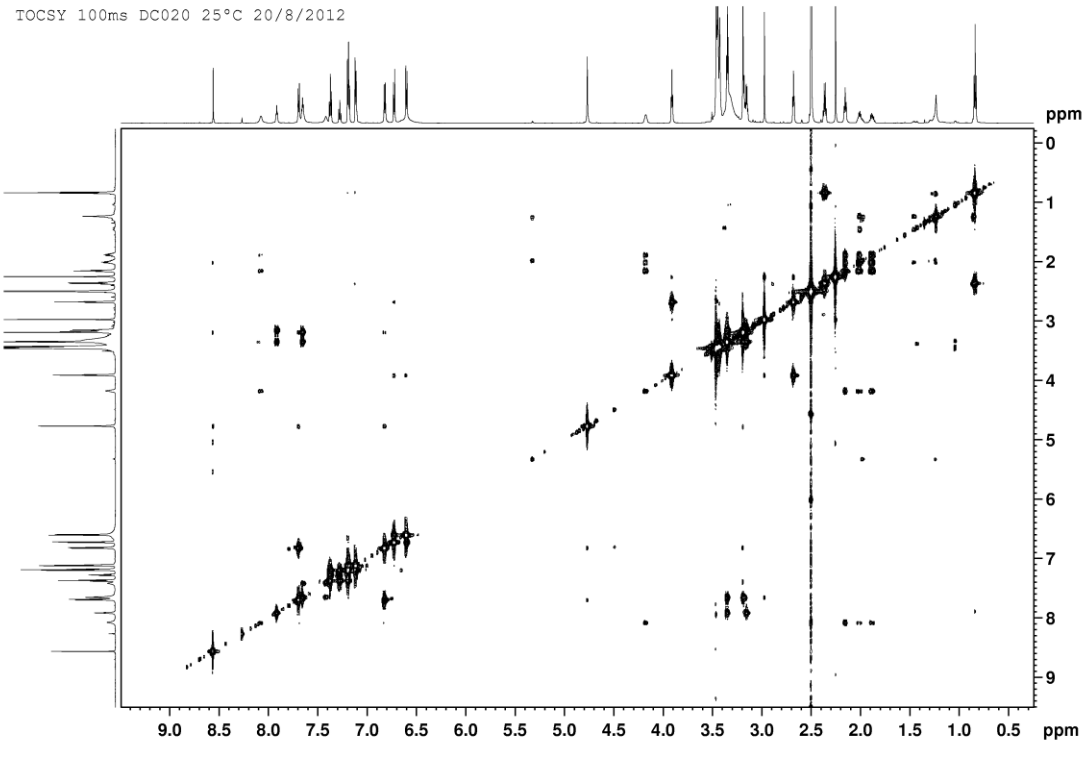

Cmpd **5** 2D ^{1}H-^{13}C meHSQC (700 MHz, DMSO-d6)

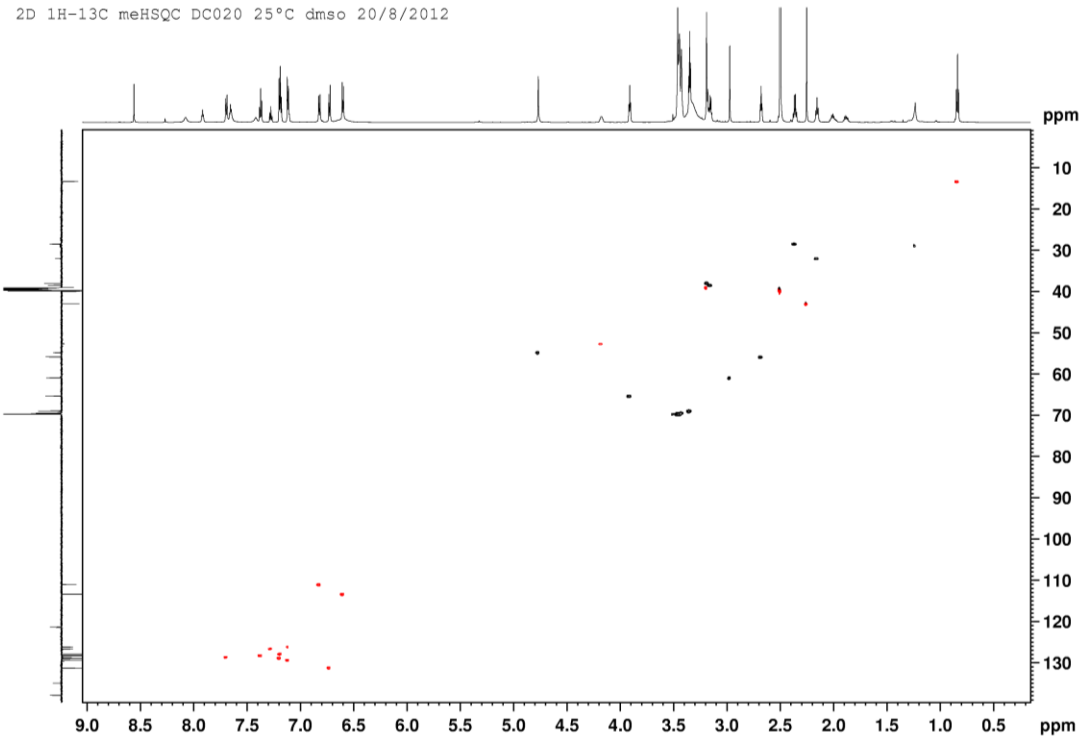

Cmpd **5** 2D ^{1}H-^{13}C HMBC (700 MHz, DMSO-d6)

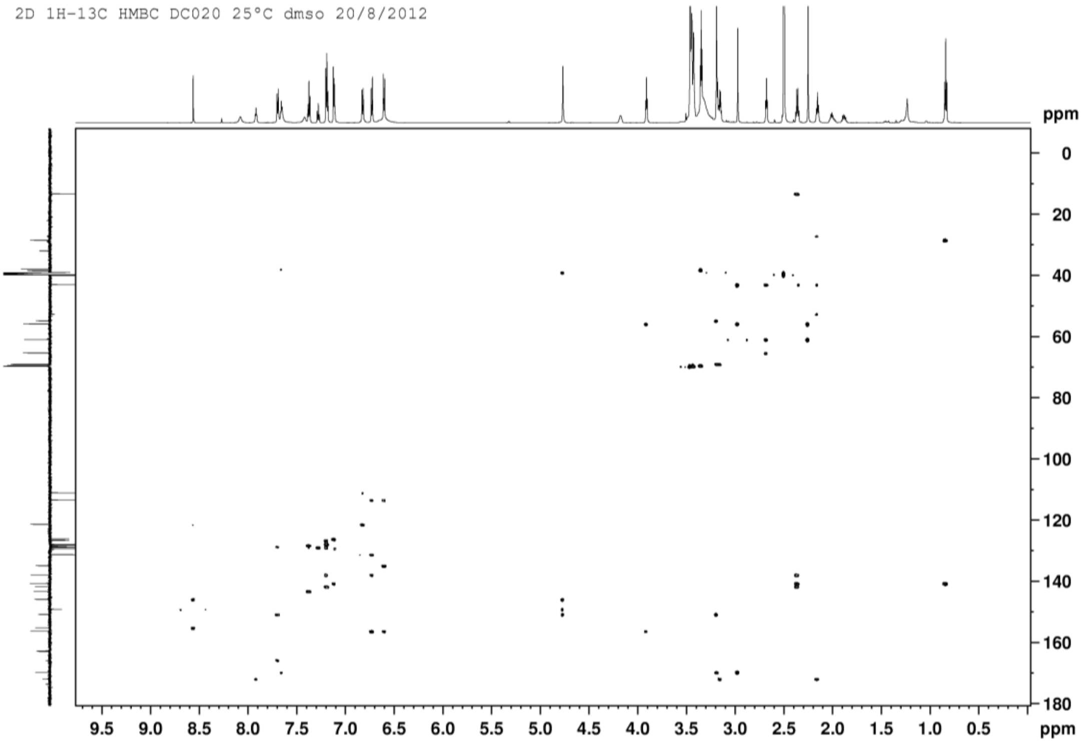

Cmpd **5** ^{13}C APT(176 MHz, DMSO-d6)

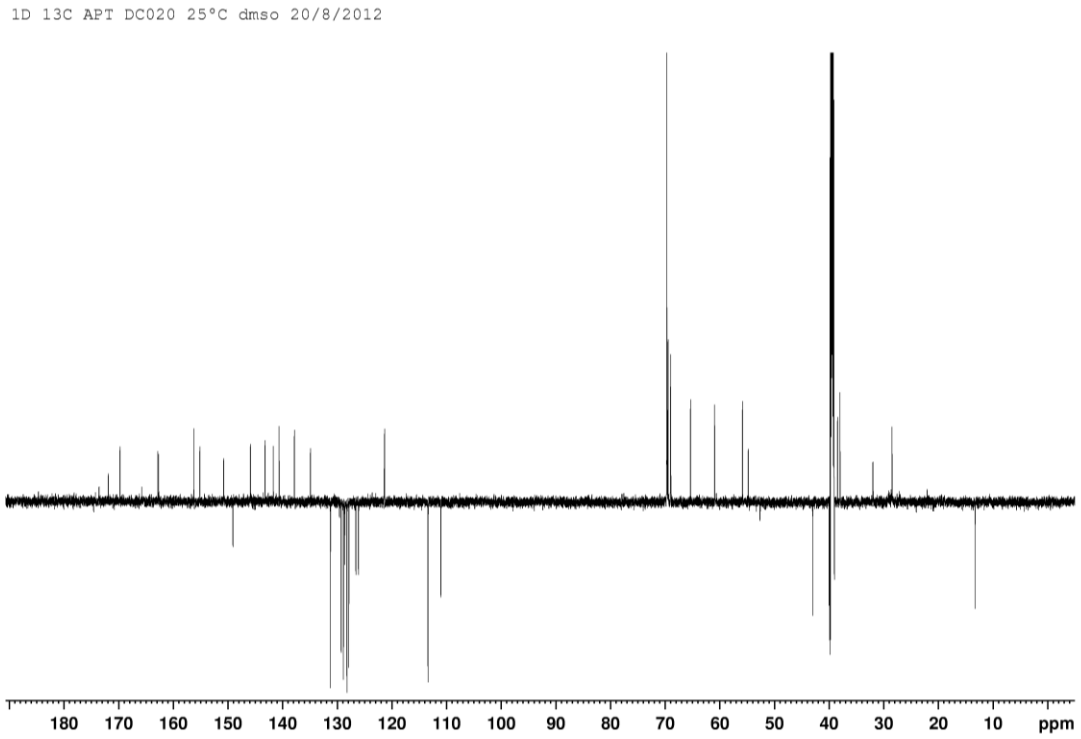

Cmpd **X** ^{1}H (700 MHz, DMSO-d6)

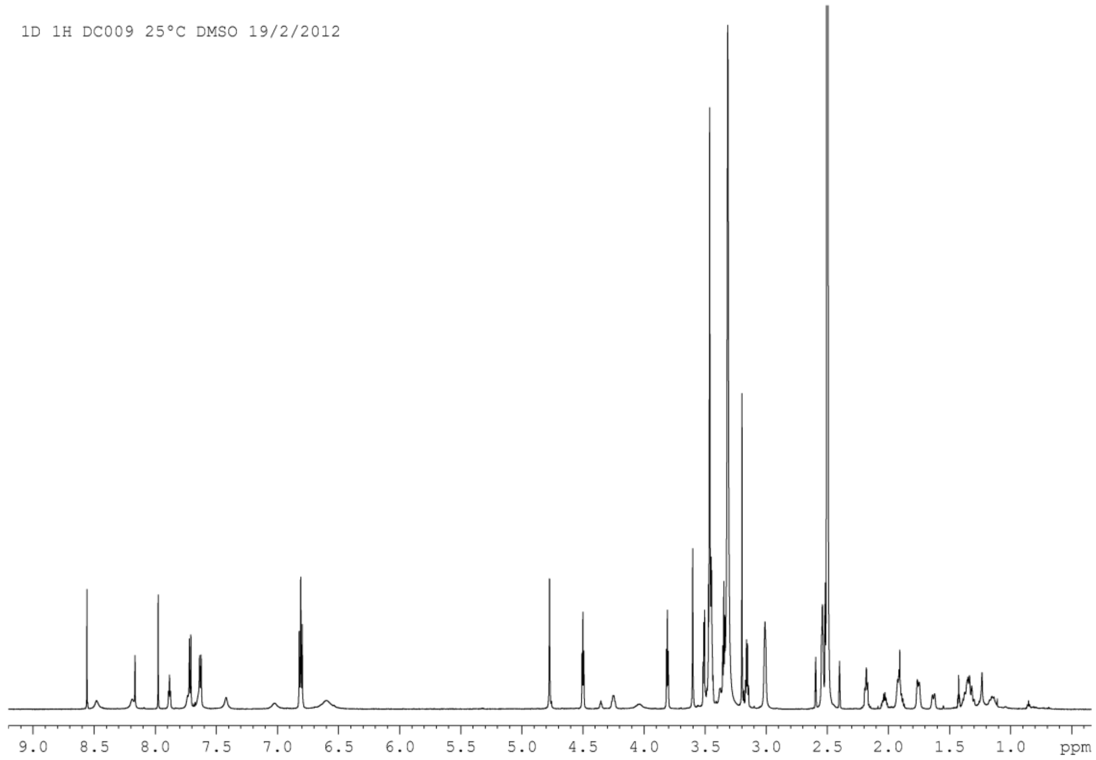

Cmpd **X** ^{1}H-^{1}H COSY (700 MHz, DMSO-d6)

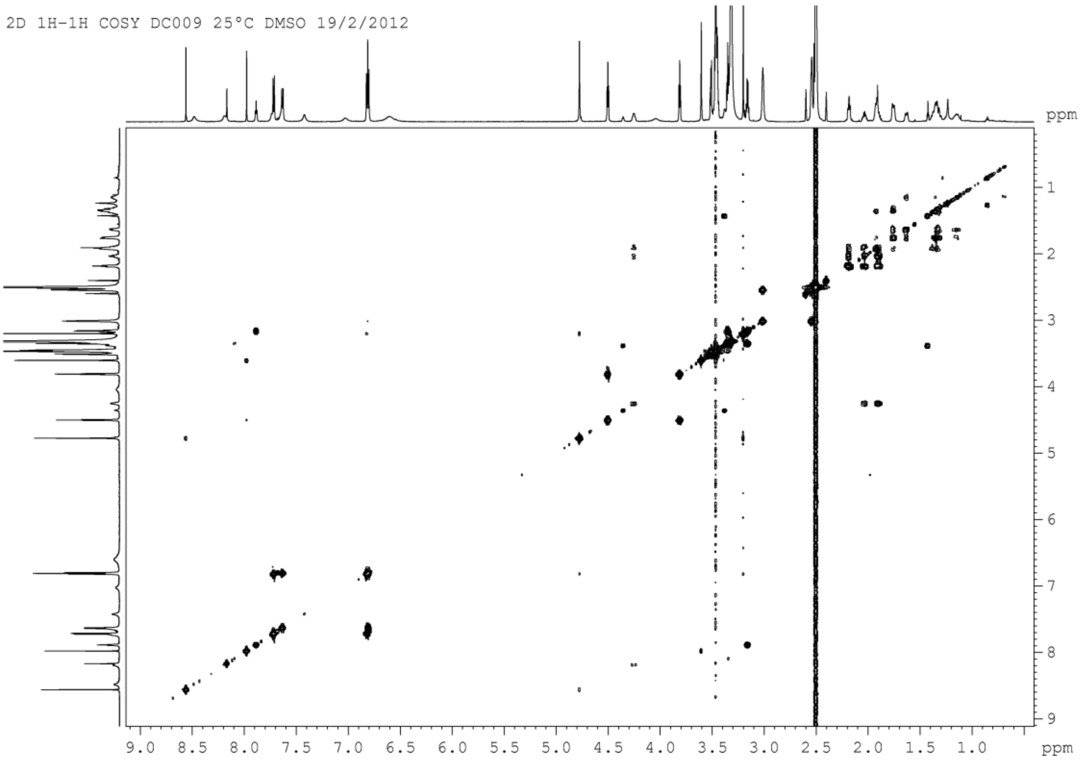

Cmpd **X** ^{1}H-1H NOESY (700 MHz, DMSO-d6)

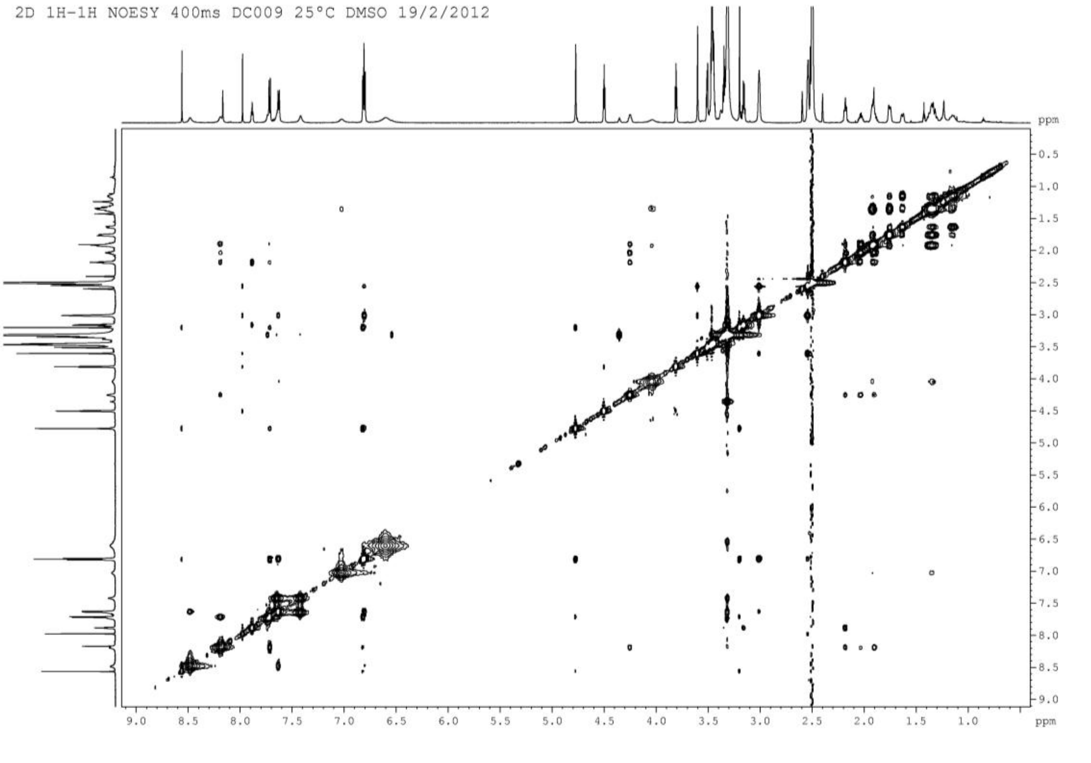

Cmpd **X** ^{1}H-^{13}C geHSQC (700 MHz, DMSO-d6)

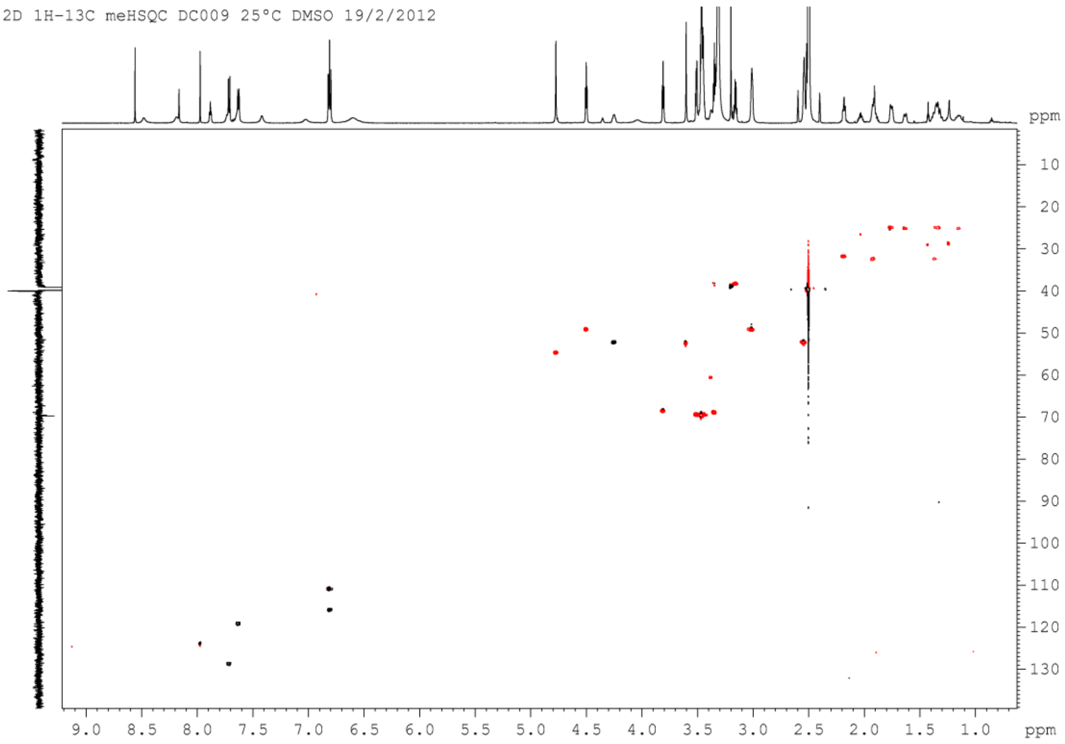

Cmpd **X** ^{1}H-^{13}C geHMBC (700 MHz, DMSO-d6)

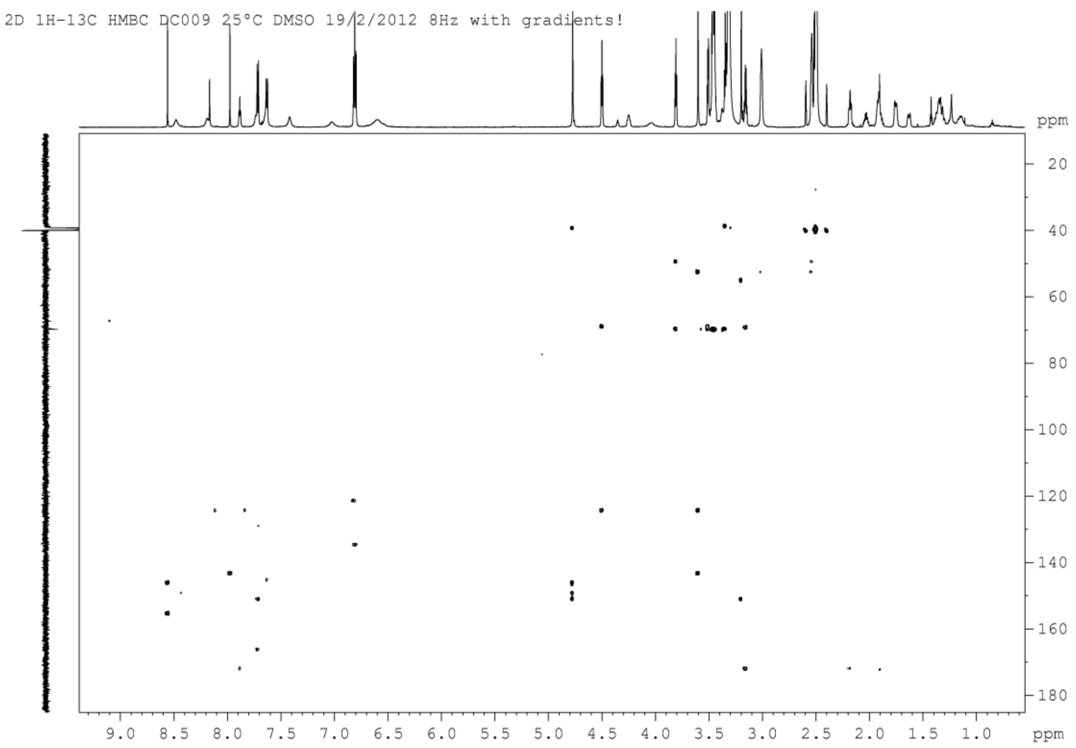

Cmpd **X** ^{1}H-^{1}H TOCSY (700 MHz, DMSO-d6)

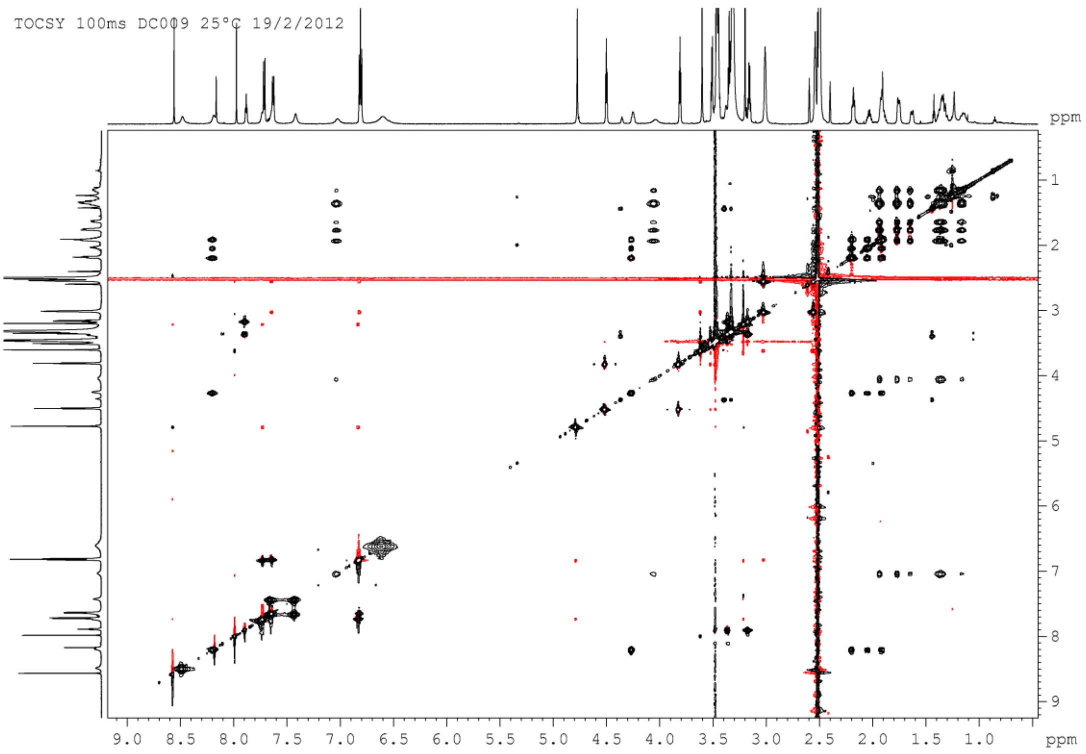

Cmpd **9** ^{1}H (700 MHz, DMSO-d6)

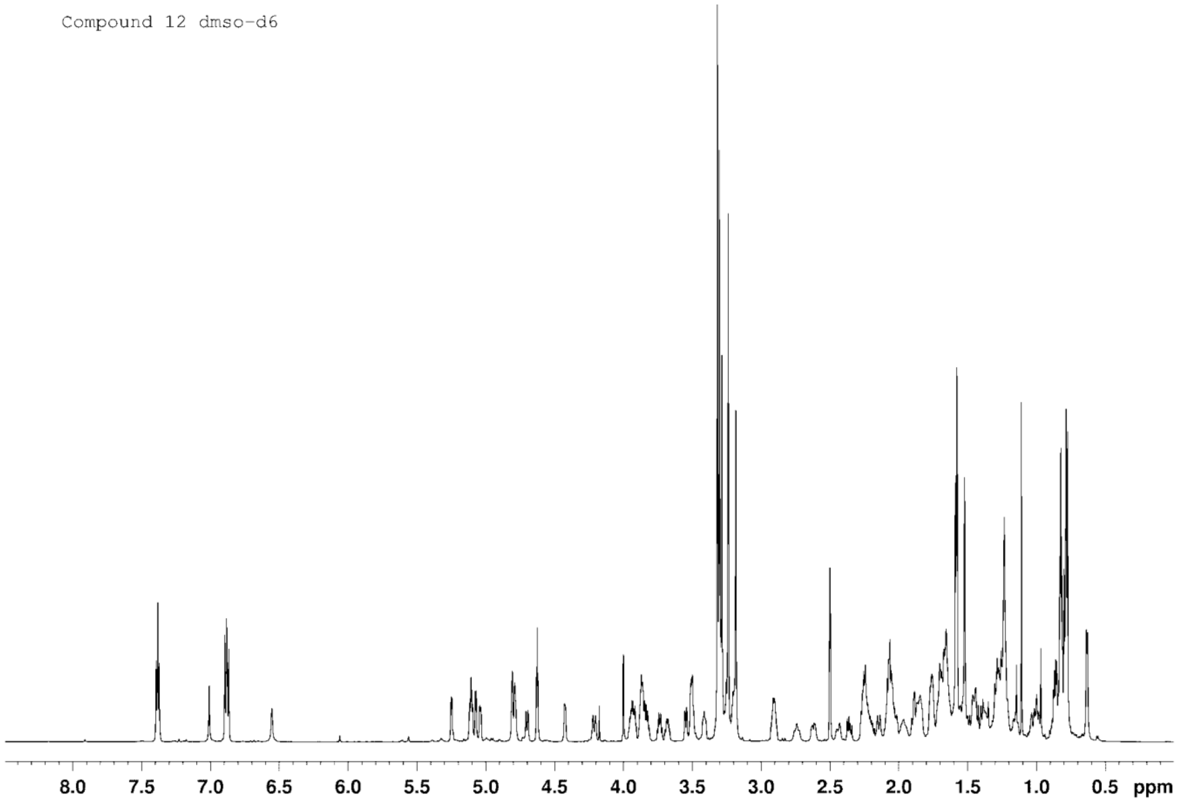

Cmpd **9** ^{13}C APT(176 MHz, DMSO-d6)

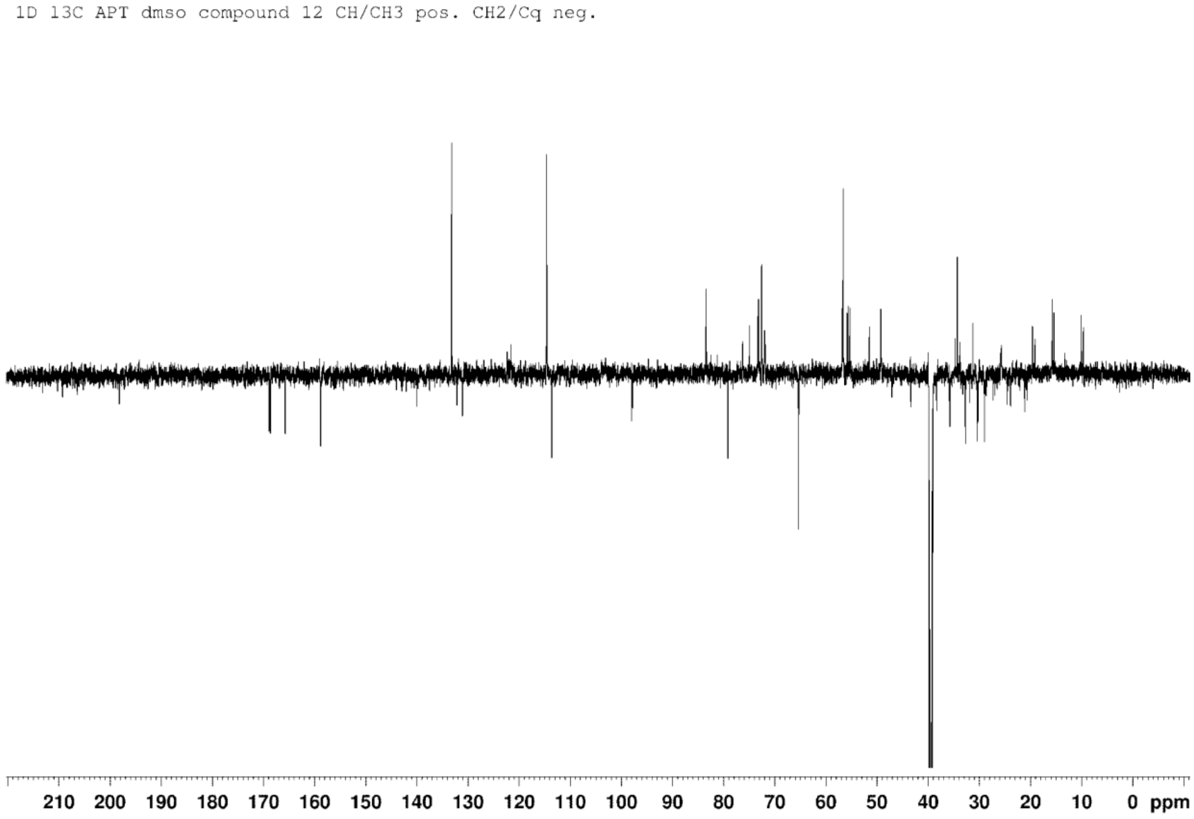

Cmpd **10** ^{1}H (700 MHz, DMSO-d6)

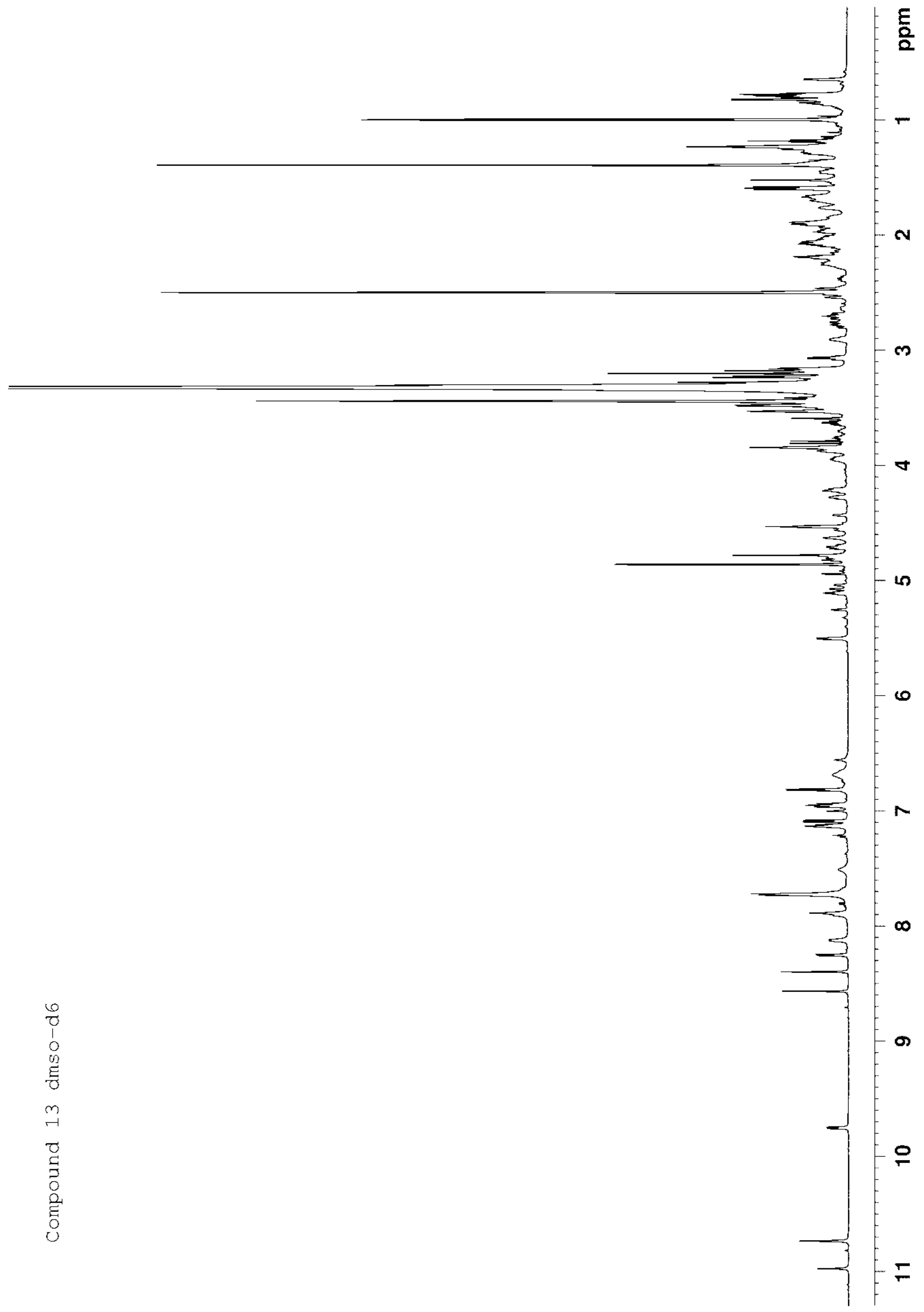

4. References

[i] Iyer, S. S.; Anderson, A. S.; Reed, S.; Swanson, B.; Schmidt, J. G. *Tetrahedron Lett.* **2004**, *45*, 4285-4288.

[ii] Francis, C. L.; Yang, Q.; Hart, N. K.; Widmer, F.; Manthey, M. K.; He-Williams, H. M. *Aust. J. Chem.* **2002**, *55*, 635-645.

[ii] Francis, C. L.; Yang, Q.; Hart, N. K.; Widmer, F.; Manthey, M. K.; He-Williams, H. M. *Aust. J. Chem.* **2002**, *55*, 635-645.

[iii] Dreaden, E. C.; Mwakwari, S. C.; Sodji, Q. H.; Oyelere, A. K.; El-Sayed, M. A. *Bioconjug. Chem.*

[iii] Dreaden, E. C.; Mwakwari, S. C.; Sodji, Q. H.; Oyelere, A. K.; El-Sayed, M. A. *Bioconjug. Chem.* **2009**, *20*, 2247-2253.

[iv] Chan, T. R.; Hilgraf, R.; Sharpless, K. B.; Fokin, V. V. *Org. Lett.* **2004**, *6*, 2853-2855.

[v] The molar amount of acid is a rough estimation given the significant amount of silica gel remaining in this material after chromatography.

[vi] Bellamy, F. D. ; Ou, K. *Tetrahedron Lett.* **1984**, *25*, 839-842.

[vii] Chen, S. ; Zhang, Q. ; Wu, X. ; Schultz, P. G.; Ding, S. *J. Am. Chem. Soc.* **2004**, *126*, 410-411.

[viii] Pruschy, M. N.; Spencer, D. M.; Kapoor, T. M.; Miyake, H.; Crabtree, G. R.; Schreiber, S. L. *Chem. Biol.* **1994**, *1*, 163-172.

[ix] Mierke, D. F.; Schmieder, P.; Karuso, P.; Kessler, H. *Helv. Chim. Acta* **1991**, *74*, 1027 – 1047.

[x] Caligiuri, M.; Molz, L.; Liu, Q.; Kaplan, F.; Xu, J. P.; Majeti, J. Z.; Ramos-Kelsey, R.; Murthi, K.; Lievens, S.; Tavernier, J.; Kley, N. *Chem. Biol.* **2006**, *13*, 711-722.

[xi] Ulrichts, P.; Peelman, F.; Beyaert, R.; Tavernier, J. *FEBS Lett.* **2007**, *581*, 629-636.

[xii] Yan, J.; Li, Q.; Lievens, S.; Tavernier, J.; You, J. *J. Virol.* **2010**, *84*, 76-87.

[xiii] Lievens, S.; Vanderroost, N.; Van der Heyden, J.; Gesellchen, V.; Vidal, M.; Tavernier, J. *J. Proteome Res.* **2009**, *8*, 877-886.

[xiv] Montoye, T.; Lemmens, I.; Catteeuw, D.; Eyckerman, S.; Tavernier, J. *Blood* **2005**, *105*, 4264-4271.

[xv] Lamesch, P.; Li, N.; Milstein, S.; Fan, C.; Hao, T.; Szabo, G.; Hu, Z.; Venkatesan, K.; Bethel, G.; Martin, P.; Rogers, J.; Lawlor, S.; McLaren, S.; Dricot, A.; Borick, H.; Cusick, M. E.; Vandenhaute, J.; Dunham, I.; Hill, D. E.; Vidal, M. *Genomics* **2007**, *89*, 307–315.
